# Supplementary material for: Associations of childhood maltreatment with hypertension in South African women: a cross-sectional study
Source: BMJ Open. 2022 Sep 19;12(9):e057436. doi: 10.1136/bmjopen-2021-057436 (PMC9486236; doi:10.1136/bmjopen-2021-057436)
Supplement: Supplementary data [file bmjopen-2021-057436supp001.pdf]

Table S1: Results of mediation analysis for the effects of childhood maltreatment on adult prevalent hypertension in a sample of South African women (N=1797)

Independent variables	Total effect			Direct effect			Indirect effect		
	Estimates	95% CI	P-value	Estimates	95% CI	P-value	Estimates	95% CI	P-value
**Any CM**	1.78	1.34-2.43	<0.001						
BMI				1.76	1.33-2.34	<0.001	1.01	0.99-1.01	0.836
HbA1c				1.78	1.35-2.39	<0.001	0.99	0.97-1.02	0.899
HIV-positive				1.77	1.32-2.38	<0.001	1.01	0.97-1.04	0.655
Current smoking				1.76	1.32-2.40	<0.001	1.01	0.94-1.08	0.826
Current alcohol				1.66	1.24-2.78	<0.001	1.07	1.01-1.15	0.036
Depressive symptom score				1.66	1.25-2.25	<0.001	1.07	1.02-1.13	0.007
ASR score				1.67	1.27-2.25	<0.001	1.06	1.01-1.12	0.024
Other traumatic exposures score				1.53	1.14-2.08	0.006	1.18	1.10-1.26	<0.001
**Multiple CM types**	1.58	1.30-1.92	<0.001						
BMI				1.56	1.29-1.89	<0.001	1.01	0.99-1.03	0.363
HbA1c				1.57	1.29-1.93	<0.001	1.00	0.97-1.02	0.948
HIV-positive				1.55	1.28-1.88	<0.001	1.01	0.99-1.10	0.283
Current smoking				1.56	1.29-1.92	<0.001	1.00	0.95-1.06	0.916
Current alcohol				1.50	1.23-1.82	<0.001	1.05	1.01-1.11	0.043
Depressive symptom score				1.55	1.23-1.82	<0.001	1.05	1.01-1.10	0.011
ASR score				1.50	1.24-1.83	<0.001	1.04	1.01-1.09	0.045
Other traumatic exposures score				1.39	1.13-1.70	0.001	1.14	1.08-1.21	<0.001
**Cumulative CM score**	1.19	1.11-1.28	<0.001						
BMI				1.19	1.11-1.28	<0.001	1.00	0.99-1.01	0.829
HbA1c				1.19	1.11-1.28	<0.001	1.00	0.99-1.01	0.500
HIV-positive				1.19	1.11-1.27	<0.001	1.00	0.99-1.01	0.422
Current smoking				1.19	1.11-1.28	<0.001	1.00	0.98-1.02	0.955
Current alcohol				1.17	1.09-1.26	<0.001	1.02	1.01-1.03	0.038
Depressive symptom score				1.17	1.09-1.25	<0.001	1.02	1.01-1.04	0.022
ASR score				1.17	1.09-1.26	<0.001	1.02	1.00-1.04	0.066
Other traumatic exposures score				1.13	1.05-1.21	0.001	1.06	1.03-1.08	<0.001

All models adjusted for age and recent rape exposure; data are odds ratios from probit models. CM, childhood maltreatment; current smoking: currently smoke any tobacco products such as cigarettes, cigars or pipes; current alcohol consumption: consumed at least one drink in an occasion within a month, respectively (one drink is equivalent to one can/bottle of beer, cider, cooler/glass of wine/tot of spirit); depressive symptom (scores): depressive symptoms during the past week using Centre for Epidemiologic Studies

Depression Scale (CES-D score); ASR: acute stress reactions measured using Davidson Trauma Scale, a validated self-rating scale. Cumulative abuse (score): ranged from 0 (no abused in any type) to 8 (often/severely abused in all 4 types).

Table S2: Results of multiple mediation analysis for the effects of childhood maltreatment on adult prevalent hypertension in a sample of South African women (N=1797)

**Independent variables**	**Total effect**			**Direct effect**			**Indirect effect**		
	Estimates	95% CI	P-value	Estimates	95% CI	P-value	Estimates	95% CI	P-value
**Any CM**	1.84	1.35-2.51	<0.001	1.35	0.96-1.88	0.084			
BMI							1.02	0.99-1.04	0.154
HbA1c							0.99	0.97-1.02	0.768
HIV-positive							1.00	0.98-1.02	0.694
Current smoking							1.01	0.95-1.07	0.867
Current alcohol							1.07	1.01-1.14	0.034
Depressive symptom score							1.05	1.01-1.10	0.040
ASR score							1.04	0.98-1.10	0.232
Other traumatic exposures score							1.14	1.07-1.23	<0.001
**Multiple CM types**	1.59	1.30-1.95	<0.001	1.26	1.01-1.58	0.045			
BMI							1.01	0.99-1.03	0.160
HbA1c							1.00	0.98-1.01	0.872
HIV-positive							1.01	0.99-1.02	0.414
Current smoking							1.00	0.95-1.05	0.947
Current alcohol							1.05	1.01-1.10	0.040
Depressive symptom score							1.04	1.00-1.08	0.067
ASR score							1.02	0.98-1.07	0.337
Other traumatic exposures score							1.12	1.06-1.18	<0.001
**Cumulative CM score**	1.19	1.11-1.29	<0.001	1.10	1.01-1.19	0.024			
BMI							1.00	0.99-1.01	0.337
HbA1c							1.00	0.99-1.01	0.364
HIV-positive							1.00	0.99-1.01	0.655
Current smoking							1.00	0.98-1.02	0.976
Current alcohol							1.02	1.01-1.03	0.038
Depressive symptom score							1.01	1.00-1.03	0.090
ASR score							1.01	0.99-1.03	0.425

Other traumatic exposures score							1.04	1.02-1.07	<0.001

All models adjusted for age and recent rape exposure; data are odds ratios from probit model. CM, childhood maltreatment; current smoking: currently smoke any tobacco products such as cigarettes, cigars or pipes; current alcohol consumption: consumed at least one drink in an occasion within a month, respectively (one drink is equivalent to one can/bottle of beer, cider, cooler/glass of wine/tot of spirit); depressive symptom (scores): depressive symptoms during the past week using Centre for Epidemiologic Studies Depression Scale (CES-D score); ASR: acute stress reactions measured using Davidson Trauma Scale, a validated self-rating scale. Cumulative abuse (score): ranged from 0 (no abused in any type) to 8 (often/severely abused in all 4 types).

Table S3: Results of mediation analysis for the effects of childhood maltreatment on adult prevalent hypertension in a sample of South African women (N=1797).

Confounder/ mediator	Average total effect			Average direct effect			Average indirect effect			Proportion of mediation effect
	Coefficient	95% CI	P-value	Coefficient	95% CI	P-value	Coefficient	95% CI	P-value	% (95%CI)
**Any CM**	0.060	0.030 – 0.090	<0.001							
BMI				0.057	0.028-0.09	<0.001	0.002	-0.000 – 0.01	0.120	4 (-0.3-14)
HbA1c				0.060	0.03-0.09	<0.001	-0.001	-0.003-0.00	0.810	-0.3 (-6.1-10)
HIV-positive				0.059	0.029-0.09	<0.001	-0.001	-0.003-0.00	0.860	-2 (-4.1-6)
Current smoking				0.059	0.03-0.09	<0.001	0.000	-0.003-0.00	0.930	0.3 (-4.9-7)
Current alcohol				0.060	0.026-0.008	<0.001	0.004	0.001-0.01	0.027	6.8 (1.5-21)
Depressive symptom score				0.054	0.024-0.008	<0.001	0.006	0.001-0.01	0.018	10 (2.2-26)
ASR score				0.055	0.026-0.09	<0.001	0.005	0.001-0.01	0.061	8.6 (0.8-28)
Other traumatic exposures score				0.046	0.013-0.08	0.009	0.018	0.011-0.03	<0.001	26.2 (15-70)
**Multiple CM types**	0.046	0.025-0.06	<0.001							
BMI				0.043	0.024-0.06	<0.001	0.002	-0.000 – 0.00	0.130	3.1 (-0.4-10)
HbA1c				0.045	0.025-0.06	<0.001	-0.000	-0.002-0.001	0.850	-0.2 (-4.2-2)
HIV-positive				0.045	0.025-0.06	<0.001	-0.000	-0.001-0.00	0.490	-0.07 (-2.7-9)
Current smoking				0.045	0.025-0.06	<0.001	-0.000	-0.002 – 0.00	0.990	-0.05 (-5.4-6)
Current alcohol				0.043	0.023-0.06	<0.001	0.003	0.000 – 0.01	0.036	6.9 (0.7-17)
Depressive symptom score				0.041	0.021-0.06	<0.001	0.005	0.001-0.01	0.026	11 (1.5-27)
ASR score				0.042	0.023-0.06	<0.001	0.003	-0.001-0.01	0.099	6.2 (-1.2-21)

Other traumatic exposures score				0.033	0.012-0.05	0.002	0.013	0.007-0.02	<0.001	30.5 (18.6-67)
**Cumulative CM score**	0.017	0.011-0.02	<0.001							
BMI				0.016	0.01-0.02	<0.001	0.000	-0.001 – 0.001	0.420	1.9 (-2.4-8)
HbA1c				0.016	0.01-0.02	<0.001	-0.000	-0.001 – 0.00	0.600	-0.7 (-5.1-1)
HIV-positive				0.016	0.01-0.02	<0.001	-0.001	-0.001-0.00	0.890	-3.1 (-10-10)
Current smoking				0.016	(0.01-0.02)	<0.001	-0.000	-0.001 – 0.001	0.910	-0.24 (-6.4-5)
Current alcohol				0.015	0.009 – 0.03	<0.001	0.001	-0.001-0.001	0.280	3.6 (-3.4-13)
Depressive symptom score				0.015	0.01-0.02	<0.001	0.002	0.000 – 0.004	0.056	10 (1-26)
ASR score				0.015	0.010-0.02	<0.001	0.001	-0.001-0.00	0.270	6.1 (-4.3-22)
Other traumatic exposures score				0.012	0.004-0.02	0.003	0.005	0.003-0.010	<0.001	30 (18-66)

All models adjusted for age and recent rape exposure. Data are regression coefficients**;** CM, childhood maltreatment; current smoking: currently smoke any tobacco products such as cigarettes, cigars or pipes; current alcohol consumption: consumed at least one drink in an occasion within a month, respectively (one drink is equivalent to one can/bottle of beer, cider, cooler/glass of wine/tot of spirit); depressive symptom (scores): depressive symptoms during the past week using Centre for Epidemiologic Studies Depression Scale (CES-D score); ASR: acute stress reactions measured using Davidson Trauma Scale, a validated self-rating scale. Cumulative CM (score): ranged from 0 (no maltreated in any type) to 8 (often/severely maltreated in all 4 types).

Table S4: Results of multiple mediation analysis for the effects of childhood maltreatment on adult prevalent hypertension in a sample of South African women (N=1797)

**Independent variables**	**Average total effect**		**Average direct effect**		**Average causal mediation effect**	
	Estimates	95% CI	Estimates	95% CI	Estimates	95% CI
**Any CM**	0.060*	0.030-0.090	0.057*	0.028-0.09		
BMI					0.003	-0.001-0.01
HbA1c					0.000	-0.001-0.00
HIV-positive					0.001	-0.001-0.00
Current smoking					-0.001	-0.005-0.00
Current alcohol					0.004	-0.003-0.01
Depressive symptom score					0.012*	0.002-0.02
ASR score					-0.011	-0.023-0.00
Other traumatic exposures score					0.016*	0.006-0.03
**Multiple CM types**	0.050*	0.028-0.070	0.038*	0.009-0.06		

BMI					0.001	-0.008-0.01
HbA1c					-0.001	-0009-0.01
HIV-positive					-0.000	-0.009-0.01
Current smoking					-0.002	-0.011-0.01
Current alcohol					0.001	-0.007-0.01
Depressive symptom score					0.005	-0.006-0.02
ASR score					-0.006	-0.017-0.00
Other traumatic exposures score					0.014*	0.002-0.03
**Cumulative CM score**	0.021*	0.012-0.03	0.024*	0.013-0.030		
BMI					-0.001	-0.001-0.01
HbA1c					-0.002	-0.008-0.00
HIV-positive					-0.002	-0008-0.01
Current smoking					-0.002	-0.008-0.01
Current alcohol					-0.001	-0.008-0.01
Depressive symptom score					-0.001	-0.008-0.01
ASR score					-0.003	-0.010-0.00
Other traumatic exposures score					0.006*	0.000-0.01

All models adjusted for age and recent rape exposure, data are regression coefficients; CM, childhood maltreatment; current smoking: currently smoke any tobacco products such as cigarettes, cigars or pipes; current alcohol consumption: consumed at least one drink in an occasion within a month, respectively (one drink is equivalent to one can/bottle of beer, cider, cooler/glass of wine/tot of spirit); depressive symptom (scores): depressive symptoms during the past week using Centre for Epidemiologic Studies Depression Scale (CES-D score); ASR: acute stress reactions measured using Davidson Trauma Scale, a validated self-rating scale. Cumulative CM (score): ranged from 0 (no maltreated in any type) to 8 (often/severely maltreated in all 4 types). *, statistical significance.

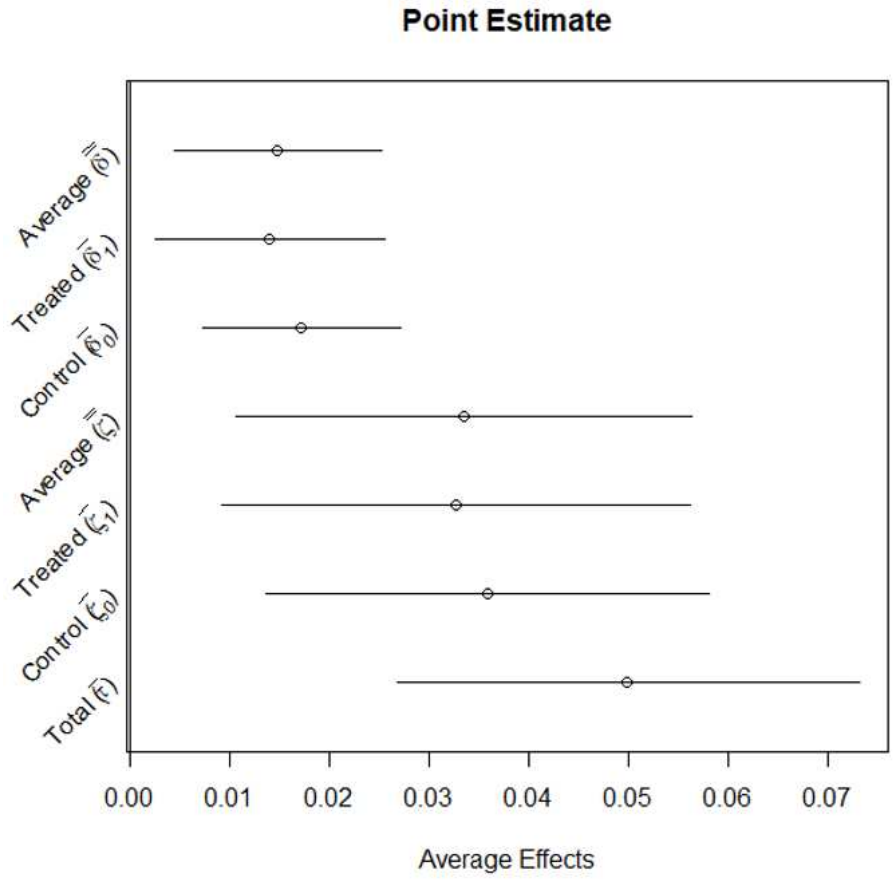

Figure S1: Graphical summary of the results from the 'multimed' function under the homogeneous interaction assumption.

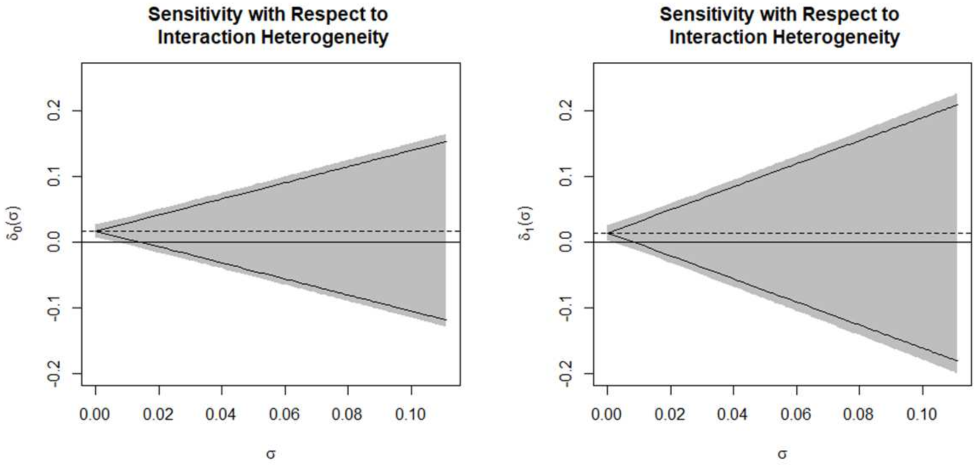

Figure S2: Graphical summary of sensitivity analysis using the 'multimed' function. Results as a function of σ and R^2. For both δ(1) and δ(0), the lower bounds sharply decrease as we increase the value of υ and cross the Zero line at small values of υ [0.011 for .δ(1) and 0.015 for .δ(0)].
