# Supplementary material for: Associations of childhood maltreatment with hypertension in South African women: a cross-sectional study
Source: BMJ Open. 2022 Sep 19;12(9):e057436. doi: 10.1136/bmjopen-2021-057436 (PMC9486236; doi:10.1136/bmjopen-2021-057436)
Supplement: Author's manuscript [file bmjopen-2021-057436.draft_revisions.pdf]

BMJ Open is committed to open peer review. As part of this commitment we make the peer review history of every article we publish publicly available.

When an article is published we post the peer reviewers' comments and the authors' responses online. We also post the versions of the paper that were used during peer review. These are the versions that the peer review comments apply to.

The versions of the paper that follow are the versions that were submitted during the peer review process. They are not the versions of record or the final published versions. They should not be cited or distributed as the published version of this manuscript.

BMJ Open is an open access journal and the full, final, typeset and author-corrected version of record of the manuscript is available on our site with no access controls, subscription charges or pay-per-view fees (http://bmjopen.bmj.com).

If you have any questions on BMJ Open's open peer review process please email info.bmjopen@bmj.com

BMJ Open

The associations of childhood maltreatment with hypertension in South African women: A cross-sectional study

Journal:	*BMJ Open*
Manuscript ID	bmjopen-2021-057436
Article Type:	Original research
Date Submitted by the Author:	15-Sep-2021
Complete List of Authors:	NGUYEN, KIM ANH; South African Medical Research Council, Non-Communicable Diseases Research Unit<br>Kengne , AP; South African Medical Research Council, Non-Communicable Diseases Research Unit; University of Cape Town Department of Medicine<br>Abrahams, Naeemah; South African Medical Research Council, Gender and Health Research Unit<br>Jewkes, Rachel; South African Medical Research Council, Gender and Health Research Unit; South African Medical Research Council, Office of the Executive Scientist<br>Mhlongo, Shibe; South African Medical Research Council, Gender & Health Research Unit<br>Peer, Nasheeta; South African Medical Research Council, Non-communicable Diseases Research Unit; University of Cape Town Department of Medicine
Keywords:	Hypertension < CARDIOLOGY, PUBLIC HEALTH, EPIDEMIOLOGY, MENTAL HEALTH, Child protection < PAEDIATRICS

SCHOLARONE™
Manuscripts

I, the Submitting Author has the right to grant and does grant on behalf of all authors of the Work (as defined in the below author licence), an exclusive licence and/or a non-exclusive licence for contributions from authors who are: i) UK Crown employees; ii) where BMJ has agreed a CC-BY licence shall apply, and/or iii) in accordance with the terms applicable for US Federal Government officers or employees acting as part of their official duties; on a worldwide, perpetual, irrevocable, royalty-free basis to BMJ Publishing Group Ltd ("BMJ") its licensees and where the relevant Journal is co-owned by BMJ to the co-owners of the Journal, to publish the Work in this journal and any other BMJ products and to exploit all rights, as set out in our licence.

The Submitting Author accepts and understands that any supply made under these terms is made by BMJ to the Submitting Author unless you are acting as an employee on behalf of your employer or a postgraduate student of an affiliated institution which is paying any applicable article publishing charge ("APC") for Open Access articles. Where the Submitting Author wishes to make the Work available on an Open Access basis (and intends to pay the relevant APC), the terms of reuse of such Open Access shall be governed by a Creative Commons licence – details of these licences and which Creative Commons licence will apply to this Work are set out in our licence referred to above.

Other than as permitted in any relevant BMJ Author's Self Archiving Policies, I confirm this Work has not been accepted for publication elsewhere, is not being considered for publication elsewhere and does not duplicate material already published. I confirm all authors consent to publication of this Work and authorise the granting of this licence.

For peer review only

The associations of childhood maltreatment with hypertension in South African women: A cross-sectional study

Kim Anh Nguyen [1], Andre Pascal Kengne [1,2], Naeemah Abrahams [3], Rachel Jewkes [3,4], Shibe Mhlongo [3], Nasheeta Peer [1,2]

[1] Non-communicable Diseases Research Unit, South African Medical Research Council, Cape Town and Durban, South Africa

[2] Department of Medicine, University of Cape Town, Cape Town, South Africa

[3] Gender and Health Research Unit, South African Medical Research Council, Cape Town and Pretoria, South Africa

[4] Office of the Executive Scientist, South African Medical Research Council, Cape Town, South Africa

Corresponding author:

Dr Kim Anh Nguyen

Postal address: Non-communicable Diseases Research Unit, South African Medical Research Council, PO Box 19070 Tygerberg, 7505 Cape Town, South Africa

Email: Kim.Nguyen@mrc.ac.za

Abstract word count # 297

Main text word count # 4330

Number of main Tables # 5

Number of Supplement Tables # 3

Number of Figures # 1

Number of References # 35

For peer review only

For peer review only - http://bmjopen.bmj.com/site/about/guidelines.xhtml

ABSTRACT

Objectives: To examine the associations of childhood maltreatment (CM) with hypertension, and the mediating effects of hypertension risk factors on the associations in South African women, using baseline data of the Rape Impact Cohort Evaluation (RICE) longitudinal study.

Design: Cross-sectional

Setting and participants: Self-reported data on CM exposure and its severity in 18-40-year-old women living in KwaZulu-Natal province were assessed. Logistic regression models, adjusted for traditional hypertension risk factors, rape exposure, HIV-infection, other traumatic exposures, depression scores and post-traumatic stress disorder (PTSD) scores, were used to examine the CM-hypertension associations.

Results: Among 1797 women, 220 (12.2%) had hypertension; the CM prevalence was marginally higher in women with hypertension than without hypertension overall (70.9% vs 57.2%) and for each abuse type: sexual abuse (20.9% vs 12.4%), physical abuse (51.8% vs 41.5%), emotional abuse (40% vs 27.6%) and parental neglect (35% vs 25.7%). Exposures to 1-2 types and 3-4 types of CM were 46.4% and 24.5%, respectively, in women with hypertension, and lower in women without (42.9% and 14.3%, respectively). Exposures to any CM (adjusted odds ratio: 1.62; 95%CI: 1.19-2.25), sexual abuse (1.64; 1.12-2.37), emotional abuse (1.57; 1.16-2.13), physical abuse (1.43; 1.07-1.92) and parental neglect (1.37; 1.00-1.86) were associated with hypertension. Exposures to an increasing number of abuse types and cumulative severity of CM overall (1.13; 1.05-1.21) and for each CM type were associated with increased odds of hypertension. Alcohol use, other trauma experienced, depression and PTSD partially mediated these associations.

Conclusion: CM was associated with hypertension; the effects were greater with multiple abuse types and severe abuse, and were partially mediated by alcohol use, depression, PTSD and other traumatic exposures. While CM must be prevented, effective mental health interventions to curb the uptake of unhealthy behaviours and the development of hypertension in women exposed to CM are key.

Key words: hypertension, childhood abuse, childhood maltreatment, sexual abuse, emotional abuse, physical abuse, parental neglect, depression, post-traumatic stress disorder, alcohol, stress

For peer review only - http://bmjopen.bmj.com/site/about/guidelines.xhtml

ARTICLE SUMMARY

Strengths and limitations of this study

- This is among few studies from low- and middle-income countries to examine the association between childhood maltreatment and hypertension.
- Blood pressures of the study participants were objectively measured during data collection, unlike other relevant studies that used a self-reported measure of hypertension only.
- Four categories of childhood maltreatment, and their frequency and severity, were assessed, which provides a comprehensive understanding of the relationship of childhood maltreatment with hypertension.
- Retrospective self-report of childhood maltreatment experiences could be subject to under reporting.
- Data were collected only in women and might not be applicable to men.

INTRODUCTION

Hypertension is highly prevalent globally with the greatest burden in sub-Saharan Africa where, at 46%, it is a major contributor to cardiovascular disease morbidity and mortality.[1 2] Together with age, obesity, unhealthy diets, physical inactivity, smoking and alcohol use, psychosocial stressors are among the established drivers of hypertension.[3] Emerging studies have identified exposures to trauma and violence as psychosocial stressors that influence hypertension risk.[4] Childhood traumatic experiences i.e. child abuse is an example of early trauma which may subsequently contribute to the development of hypertension.[5]

Childhood maltreatment (CM) such as sexual abuse, physical abuse, emotional abuse and parental neglect have increasingly been reported in many parts of the world where at least half of the children were exposed to these traumas.[6] The overall prevalence estimated ranges from 12% reported in Europe, to 50% in Africa and 64% in Asia. The authors warn the wide variations across regions may be due time of survey, types of CM assessed, and definitions used. The psychological impact of CM is well-established with CM-induced traumatic stress contributing to depression, anxiety, post-traumatic stress and maladaptive risky lifestyle behaviours across lifespan.[7–9] Growing evidence suggested that CM also adversely impacts

For peer review only - http://bmjopen.bmj.com/site/about/guidelines.xhtml

physical health, resulting in a wide range of disorders. These include coronary heart disease, obesity, asthma and autoimmune diseases, among other conditions.[7 10]

Although extensive research has found that chronic psychosocial strain contributes to the development of hypertension overtime,[4] and CM has been considered such a stressor,[5] the current evidence on this relationship is inconsistent. Some studies have demonstrated that CM, particularly sexual abuse and physical abuse, were associated with hypertension.[11] Other studies, however, have shown no such association[12] or only an indirect association via psychological disorders or through the link with obesity.[11] However, these findings were from a few studies conducted mostly in developed countries, with variations in methodologies especially in measurements and definitions of CM as well as the scope of CM investigated.[11–13]

South Africa has high burdens of both hypertension[14] and CM,[15–17] calling for the exploration of an association, if any, between these two factors. Therefore, this study aimed to examine the associations of CM including sexual abuse, physical abuse, emotional abuse and parental neglect with the presence of hypertension in 18-40-year-old South African women, using the baseline data of the Rape Impact Cohort Evaluation (RICE) longitudinal study.[18] Additionally, we explored the mediating effects of potential hypertension risk factors such as body mass index (BMI), glycated haemoglobin (HbA1c), HIV-infection, rape exposure, current smoking, current alcohol consumption, current depressive symptom, post-traumatic stress disorders (PTSD) and other traumatic exposure on the associations.

METHODS

Study design and population

The RICE cohort study was conducted in the Greater Durban Metro area in the KwaZulu-Natal province from October 2014 to March 2020. The study included rape exposed participants (n=947) recruited from five public health-based post-rape service and non-rape exposed participants (n=852) (controls) recruited from adjacent public health primary healthcare clinics. The control and rape exposed women were likely to be similar because they were recruited from the same geographical area and were of similar ages to ensure comparability.[18 19] The details of the study protocol has been published previously.[18] Eligible participants for both arms were women aged 18-40 years while those who were more than 14 weeks

For peer review only - http://bmjopen.bmj.com/site/about/guidelines.xhtml

pregnant, lactating, had cognitive disabilities or severely mentally distressed rape survivors were excluded. Further exclusion among women in the control arm were those who reported lifetime exposure to rape or force sex. Furthermore, the baseline interviews were done within 20 days of the incident rape event among the rape-exposed participants.

Data collection

The data collection was done using an electronic data capturing and management system (Bryant System). Trained fieldworkers administered questionnaires, clinical examinations and biochemical assessments. Interview data collected included socio-demographic variables (age, education level, employment status and residence type and area), medical history, lifestyle behaviours (alcohol and tobacco use), exposure to the different childhood exposure types and mental health assessments (depressive symptoms, post-traumatic stress disorder).

Measurements and definitions

Socio-demographic and lifestyle characteristics:

Education level was categorized as primary (1-7 school years), high-school (8-12 school years) and higher; while residence was classified as formal urban, informal urban, rural and semi-rural. Current alcohol consumption was defined as consumed at least one drink within a month (one drink equivalent to one can/bottle of beer, cider, cooler/glass of wine/tot of spirit); current smoking as "currently smoke any tobacco products such as cigarettes, cigars or pipes".

Childhood maltreatment and mental health outcomes:

CM was assessed using the Childhood Trauma Questionnaire Short Form (CTQ-SF) scale which was adapted and used previously in South Africa.[8 20] Thirteen items were used to measure four abuse types: sexual abuse (4 items), emotional abuse (3 items), physical abuse (3 items), and parental neglect (3 items). Participants' exposure to each of these items before 18 years of age was determined. Exposures were evaluated as yes/no for each type of abuse followed by the frequency of exposure (never (score 0), some (score 1), often (score 2)). Overall CM was defined as exposure to any of the four abuse types and categorized as no exposure (coded 0) and exposure to any abuse (coded 1). Exposure to multiple combined CM types was coded

For peer review only - http://bmjopen.bmj.com/site/about/guidelines.xhtml

as none (coded 0), 1-2 types (coded 1), and 3-4 types (coded 2). The sum of the frequency of exposure for all CM types described the cumulative severity. The scores ranged from 0 (no abuse) to 8 (severe or frequent abuse).

Post-traumatic stress disorder/Acute stress reactions (ASR)

PTSD are acute stress reactions to a traumatic event that are sustained beyond 1-month post the event. The baseline assessment among the rape exposed participants were done within 20 days of the incident rape and PTSD due to the rape can therefore not be ascertained and referred to as acute stress reactions.[21] The validated Davidson Trauma self-rating scale was used to measure PTSD/ASR which included 30 items.[22] Participants were asked how much each item/symptom affected them over the past week, with responses evaluated on a 5-point Likert scale: “not at all” (0), “only once” (1), “2-3 times” (2), “4-6 times” (3) and ‘all the time’ (4). Scores for all items were summed for a PTSD score.

Current depressive symptoms were measured using the 20-item Center for Epidemiologic Studies Depression (CESD) self-report measure.[23] The CESD scale was designed to assess how often a person experienced depressive symptoms within the past week. The response was categorized into a 4-point Likert scale ranging from “rarely or none of the time” (0) to “most or all of the times” (3). The summed score provided the overall CESD score with higher scores indicating more severe depressive symptoms.

Exposures to other prior traumatic events were assessed using an adapted Life Event Checklist.[24] The checklist includes 10 items of traumatic events namely imprisonment, civil unrest/war, serious injury, being close to death, witnessing a murder of family or friend, unnatural death of family or friend, witnessing the murder of stranger, torture, robbed or carjacked at gun or knife point and kidnapping. The yes (score 1)/no (score 0) scores for all the items were totaled to create a continuous variable of “other traumatic exposures”. A higher score was indicative of more lifetime traumatic events or exposures.

Clinical parameters

Anthropometric measures were done using standardized methods. Height was measured to the nearest millimetre with the participant in an upright position and bare-footed. Weight was taken to the nearest 0.1 kg with the participants in light clothing and without shoes. BMI

For peer review only - http://bmjopen.bmj.com/site/about/guidelines.xhtml

was calculated as weight in kilograms divided by height in metres squared, and BMI ≥25kg/m^2 was used to define overweight and obese. Waist circumference (WC) was recorded to the nearest millimetre at the level that is mid-way between the upper border of the hip bone and the lower border of the lowest rib. Elevated WC was defined as WC >80cm.[25]

Blood pressure (BP) was measured using a digital BP monitor (Omron, M6 Comfort, Netherland) after the participant was seated in a resting position for at least five minutes; three measurements were taken three minutes apart, and the average of the 2nd and 3rd readings were used in the analysis. Hypertension was defined as systolic BP ≥140mmHg or diastolic BP ≥90mmHg or self-reported previous diagnosis of hypertension.[3]

Biochemical parameters

Biochemical measurements were done at an accredited pathology laboratory (Global Laboratory, Durban, South Africa). Serum lipids were analysed by enzymatic colorimetric method; HbA1c was measured using high-performance liquid chromatography (VARIANT II TURBO. EDTA tubes) following the National Glycathaemoglobin Standardisation Programme (NGSP). Dyslipidaemia was based on the South African national lipid guidelines and defined as follows: total cholesterol >5 mmol/L, low-density lipoprotein cholesterol >3 mmol/L, triglycerides >1.5 mmol/L and high-density lipoprotein cholesterol <1.2 mmol/L.[26] Dysglycaemia was defined as HbA1c ≥5.7% and or previously diagnosed with diabetes.[27]

Statistical analyses

Data were analyzed using R statistical software version 3.6.0 (2019-04-26). Although the RICE study is a cohort study with two arms, this analysis used the baseline data from the pooled group of women and not just the rape exposed women. Exploratory analyses revealed no significant interaction effects of 'rape exposure' on the association of CM with status for hypertension, motivating the pooled analyses of both rape exposed and unexposed women to maximize the statistical power.

The baseline characteristics were summarized as means (standard deviation, SD) or medians (25th to 75th percentiles) for continuous variables, and as count (percentages) for categorical variables in the overall sample and by hypertension status. The relations of CM with hypertension were explored across 1) each of the four CM categories, 2) multiple combined

For peer review only - http://bmjopen.bmj.com/site/about/guidelines.xhtml

CM categories and 3) cumulative frequency and severity of CM. The associations were explored using multiple logistic regression analyses with adjustments for potential confounders and mediators. The potential confounders were identified as those variables which were likely correlated with CM and causally linked to the development of hypertension and included the recent rape exposure, education level, employment status and residence type. Mediators were identified as those variables which were likely involved in the pathways of the relationship between CM and hypertension, and included BMI, HbA1c, HIV-infection, current smoking, current alcohol use, depression scores and PTSD scores. The covariates of age and other traumatic exposure were also adjusted for in the models. Variables not found to be associated with hypertension in the bivariate analysis ($p > 0.05$) were subsequently excluded from the regression models. The latter included education level, employment status and residence type.

The mediation effects of the variables adjusted for in the regression models on the association of CM (any CM, multiple combined CM types and the cumulative severity score) with hypertension were explored using simple mediation analysis.[28] Each mediator was analysed separately in individual mediation models, which comprised the hypertension outcome variable, a predictor (CM exposure) and a mediator. *Laavan* package (http://CRAN.R-project.org/package=lavaan) was used to compute the indirect, direct and total effects estimates, and proportions of mediation effects (indirect effect/total effect), with the significance of the mediation effect tested via bootstrap methods, based on 5000 replications. Estimates are reported as standardized beta coefficients which were derived based on diagonal weighted least square method, but also as odd ratios (OR) and 95% confidence intervals. It is of note, however, that the interpretation of OR from probit models (implemented for mediation analysis in *Laavan*) is not straightforward. We further explored the mediating effects using multiple mediation model with adjustments for potential confounders (age, rape exposure). A p-value <0.05 was considered a statistically significant mediation effect. Full or complete mediation is present when the total and indirect effects are significant while the direct effect is non-significant. Partial mediation occurs when the total and indirect effects are significant, and the direct effect remains significant.

Patient and public involvement

For peer review only - http://bmjopen.bmj.com/site/about/guidelines.xhtml

In this study, the research questions and outcomes were not informed by patients' priorities, experiences, and preferences. Patient and the public were not involved in the design or conduct or reporting or dissemination plan.

RESULTS

A total of 1799 women were recruited into the study. Two participants had missing data on CM and were excluded. Therefore, the final sample consisted of 1797 participants. Of these, 220 (12.2%) had prevalent hypertension. The characteristics of the participants are presented in Table 1. The overall median age was 24 years; participants with hypertension were older compared to their counterparts without hypertension (26 years vs. 24 years, $p<0.001$). Compared to those without hypertension, women with hypertension were more likely to be HIV-positive and to currently drink alcohol; they had higher scores for depression, PTSD/ASR, and were more likely to have experienced other traumatic events in their lives (all $p \leq 0.05$). Participants with hypertension had higher mean WC and BMI levels and dysglycaemia, but their lipid profiles were similar to those without hypertension. There was no difference in terms of education, residence, current rape exposure or smoking status between the participants with and without hypertension (Table 1).

Characteristics of CM items by hypertension status

Table 2 shows that the participants experienced a range of abuse across the four abuse types with all abuse experiences more common in participants with, than without, hypertension, although the differences were not always significant. The most common sexual abuse inflicted was *'Someone touched my buttocks or genitals or made me touch them when I did not want to'* (hypertension vs. no hypertension: 11.8% vs. 7.8%, p=0.058). Physical abuse of '*Being beaten at home with a belt or stick or whip or something else which was hard'* was the most common abuse reported and significantly higher in women with, than without, hypertension (46.4% vs. 39.0%, p=0.043). The most frequent emotional abuse of '*Being told was lazy or stupid or weak by someone in the family'* was significantly higher in women with (28.2%), than without (16.5%), hypertension ($p<0.001$). The most common form of parental neglect, where participants '*lived in different households at different times'* during their childhood was 28.6% in women with and 21.5% in women without hypertension (p=0.021)

For peer review only - http://bmjopen.bmj.com/site/about/guidelines.xhtml

For peer review only

Prevalence of CM types by hypertensive status

The distribution of CM types overall and by hypertension status is presented in Table 3. The prevalence of CM was 58.9% in overall sample, with physical abuse being the most frequent (42.7%). This was followed by emotional abuse and parental neglect at 29% each, while exposure to sexual abuse was 13.4%. The prevalence of all four CM types was significantly higher in participants with, than without, hypertension. The prevalence of CM was 70.9% in women with vs. 57.2% in those without hypertension ($p<0.001$). More than half the women with hypertension (51.8%) had experienced physical abuse compared to 41.5% of women without hypertension ($p=0.004$). A fifth (20.9%) vs. 12.4% of women with compared to without hypertension were exposed to sexual abuse in childhood. Exposures to 1-2 types and 3-4 types of CM were 46.4% and 24.5%, respectively, in women with hypertension, and lower in women without (42.9% and 14.3%, respectively) ($p<0.001$). Similar patterns of higher prevalence rates in women with compared to without hypertension were observed for the severity and frequency of abuse experienced (all $p\leq0.003$). There was no evidence of statistically significant interaction effect of rape exposure on the distribution of CM by status for hypertension as shown in Table 4 (all interaction $p\geq0.061$).

Associations of CM exposure with prevalent hypertension

The odds ratios for the relationship of CM with hypertension adjusted for age, BMI, current smoking, current alcohol use, recent rape exposure, HIV infection, other trauma exposure, depression scores, PTSD scores and dysglycaemia are presented in Table 4. There was a positive association of exposure to any CM (adjusted odds ratio (AOR): 1.62; 95%CI: 1.19-2.25), sexual abuse (AOR: 1.64; 1.12-2.37), physical abuse (AOR: 1.43; 95%CI: 1.07-1.92) and emotional abuse (AOR: 1.57; 1.16-2.13) with prevalent hypertension. The association of exposure to parental neglect (AOR: 1.37; 1.00-1.86) was borderline. Increasing number of maltreatment types as well as cumulative severity/frequency of maltreatment overall or by maltreatment type were associated with increased odds of hypertension (Table 4).

The results of the mediation analysis showed that the associations of CM (any CM, multiple combined CM types and the cumulative severity score) with hypertension were partially mediated by current alcohol consumption (all $p\leq0.043$ for the mediation effects), depressive symptom score (all $p\leq0.022$ for the mediation effects) and experiencing other traumatic

For peer review only - http://bmjopen.bmj.com/site/about/guidelines.xhtml

events in life (all $p<0.001$ for the mediation effects) (Table 5 and Table S1). PTSD score partially mediated the association of any CM with hypertension ($p=0.024$ for the mediation effect) and of multiple CM types ($p=0.045$ for the mediation effect) but not of the cumulative severity abuse score ($p=0.066$ for the mediation effect). BMI, HbA1c, HIV status, smoking habit or recent rape exposure had no significant mediation effects on the association between CM exposure and hypertension (all $p\geq0.144$ for the mediation effects).

When the multiple mediation analysis model adjusted for age and rape exposure were applied (Tables S2 and S3), the direct effects of any CM ($p=0.084$ for the direct effect) were completely mediated by current alcohol consumption ($p=0.034$ for mediation effect), depressive symptom score ($p=0.040$) and experiencing other traumatic events ($p<0.001$). The direct effects of multiple CM types and the cumulative severity abuse score on hypertension remained significant, and the associations were partial mediated by current alcohol consumption and experiencing other traumatic events (Figure 1).

DISCUSSION

This study confirms CM is highly prevalent and associated with hypertension in South African adult women attending specific healthcare services. To our knowledge, this is among very few studies conducted in low- and middle-income countries, which describes significant associations between CM and hypertension in women after adjusting for traditional hypertension risk factors, other traumatic exposures and mental illness. The study showed exposure to sexual, physical, emotional abuse, multiple (3-4) abuse types and frequent and severe exposure were significantly associated with hypertension. Further it showed that the associations were potentially mediated by alcohol use, other trauma exposure, depression, and PTSD.

These findings accord with the literature from high-income countries; frequent and severe exposure to physical, emotional and sexual abuse and parental neglect in childhood, and exposure to multiple abuse types compared to lesser abuse exposures may have greater negative health consequences,[29] including hypertension.[30,31] The latter includes two longitudinal studies from the United States. Among 394 men and women followed for 23-years, those with multiple CM exposures showed a greater increase in adult BP levels compared to those without childhood traumatic experiences.[30] The Nurses' Health Study II

For peer review only - http://bmjopen.bmj.com/site/about/guidelines.xhtml

showed that severe childhood physical and/or sexual abuse was associated with an increased risk of hypertension in adulthood independent of race, oral contraceptive use, BMI, smoking, alcohol use, exercise and child somatogram score.[31]

However, these two studies, unlike the current study, did not investigate the effects of psychological factors such as depression, anxiety, PTSD on the relationship between CM and hypertension. Notably, the current study found that the associations of CM with hypertension were independent of psychological factors for all abuse types. Exposures to CM may independently activate the hypothalamic-pituitary-adrenal axis and the sympathetic nervous system and promote inflammation, similar to the postulated pathways for other stressors.[4] These physiological changes increase the production of hormones such as catecholamine and glucocortisone which raise BP levels. The repeated activation of these systems with multiple or frequent exposures to CM may lead to sustained increases in BP and contribute to the development of hypertension.[4] Current depressive symptom, ASR/PTSD and other prior traumatic events mediated the relationships between CM and hypertension in this study. The additive influences of these psychological factors likely demonstrate their role in the development of hypertension. While a detailed exploration of these associations is beyond the scope of this paper, the World Mental Health Survey reported similar findings. The presence of three or more childhood adversities (abuse, neglect, other trauma experiences) was modestly associated with self-reported hypertension, with the association not fully explained by early-onset or current depression-anxiety.[32] In addition, a survey in Brazil found childhood physical abuse and family violence were associated with a greater likelihood of adult hypertension; the association was attenuated by the onset of depression in adulthood. [13] Although CM was associated with hypertension independent of current mental health disorders in the current study, it is important to investigate whether the association is reversible. Such understanding will help to design interventions to prevent the development of hypertension in the CM victims.

While exposures to any CM, sexual abuse and emotional abuse were significantly associated with hypertension, there was weaker effect of physical abuse or parental neglect with hypertension in the present study. More research, particularly from longitudinal studies, is required for a deeper understanding of these associations and to examine the differential impact of the various types of CM on the development of hypertension. Similar to these

For peer review only - http://bmjopen.bmj.com/site/about/guidelines.xhtml

findings, a prospective childhood study in the United States reported no significant differences in the associations of physical abuse and neglect with the development of hypertension after 30 years of follow-up.[33] However, unlike the current study, they also found no association between childhood sexual abuse and hypertension. The study controlled for age, gender and race but did not assess the severity of CM.

Another study that examined childhood physical and verbal abuse and neglect using a short-form family environment questionnaire found an indirect association of adverse family environment in childhood with changes of BP over a ten-year period through negative emotions of depression, anxiety or anger.[34] The latter study is not directly comparable to the current study because the questionnaire used only partially addressed verbal and physical abuse and neglect with these assessed as a composite variable.

Additionally, alcohol use partly mediated the relationship between CM and hypertension in the present study. This is in keeping with a systematic review that suggested the association of CM with hypertension may also be influenced by the traditional behavioural risk factors for hypertension.[7] The uptake of risky behaviours were suggested to be coping mechanisms when faced with stressful situations.[5] However, apart from alcohol intake, other traditional risk factors for hypertension such as adiposity, assessed using BMI, did not mediate the associations of CM with hypertension in the RICE study. This may be because the participants in our study were relatively young. Furthermore, our sample was on average overweight-to-obese, restraining the distribution of markers of adiposity in this population. This in turn could affect any reliable investigation of the relationship of adiposity with hypertension in this sample.

Strengths and limitations

The BP of study participants was objectively measured during data collection, unlike other relevant studies that used a self-reported measure of hypertension only.[13 31 32] This study, therefore, likely captured all participants with hypertension instead of only those who were aware of their hypertension status. Another strength is that this study comprehensively assessed CM by examining four CM types as well as the frequency and severity of the abuse using a well-established locally validated measure.[8] Our study is one of few studies that has assessed the associations of multiple CM types and their frequency and severity with

For peer review only

For peer review only - http://bmjopen.bmj.com/site/about/guidelines.xhtml

hypertension. This paper, therefore, provides a comprehensive understanding of the relationship between CM and adult hypertension.

The present study has several limitations. The cross-sectional design precludes a reliable quantitative interpretation of the effect of the exposure of interest, the mediation effect and the inferences on causality. However, although the date of hypertension onset is unknown, but the prevalence is current, and CM occurred in the past, it is likely that this study may have established the temporal relationships between CM and hypertension. It is also known that retrospective self-report of CM experienced could be subject to under reporting due to suppressed memory.[35]

Furthermore, BP was measured only on a single occasion, which is not a formal diagnosis of hypertension and tends to over-estimate hypertension. Another limitation is that the volunteer sample recruited do not represent all women who are raped or those who seek care post rape. Similarly, the control sample of volunteers recruited from primary health care services do not represent the general population of women. However, we assumed the two groups to be similar in that they were both active health service users. The data were collected only in women and might not be applicable to men. The non-significant influences of BMI on the associations of CM with prevalent hypertension need to be viewed with caution. It could be that our study population was young (median age 24 years, 25th-75th: 21-29), in whom the traditional risk factors may yet influence the development of hypertension. Considering that the RICE study is a longitudinal study, the follow-up data may demonstrate the influence of traditional risk factors on the associations of CM with hypertension later in the life of these women.

CONCLUSIONS

CM is a serious public health problem and associated with adult hypertension independent of traditional risk factors, other traumatic events, and mental ill-health in young South African women. The associations of CM with hypertension were found to be greater in women with multiple maltreatment types and frequent and severe maltreatment, and were partially mediated by alcohol use, current depression, PTSD and other traumatic exposures. These findings suggest that prevention of CM is important, not only in itself, but also for the prevention of adult hypertension. Where the prevention of CM is not possible, counselling

For peer review only - http://bmjopen.bmj.com/site/about/guidelines.xhtml

to manage stress and improve coping skills are important to curb the development of hypertension. This is in line with the goals of psychological therapy for CM survivors which aim to reduce the subsequent health impact. Screening for hypertension in individuals suffering from CM may help early identification and treatment of the condition. Further, counselling for the other traumatic exposures, and management of depressive symptoms and PTSD are also required and may contribute partially to curbing hypertension.

Contributors: KAN led the analysis and drafted the paper. NP, NA, RJ and APK co-conceived and designed the paper. APK, NP, NA, RJ and SM co-conceived the broader study, commented on all drafts, and approved the final submission. APK assisted with the statistical analysis.

Funding: The RICE study was funded by South African Medical Research Council: Flagships Awards Project SAMRC-RFA-IFSP-01-2013. The work reported herein was made possible through funding by the South African Medical Research Council through its Division of Research Capacity Development under the SAMRC Postdoctoral Programme from funding received from the South African National Treasury. The content hereof is the sole responsibility of the authors and do not necessarily represent the official views of the SAMRC or the funders.

Competing interests: The authors declare no conflict of interest.

Patient consent for publication: Not required.

Ethics approval: The South African Medical Research Council Research Ethics Committee provided ethical approval for this study (SAMRC; EC019-10/2013), and all participants provided informed consent.

Data sharing statement: Data are available on request.

REFERENCES

1. World Health Organization. A global brief on hypertension : silent killer, global public health crisis: World Health Day 2013. World Heal. Organ. 2013;:40.https://apps.who.int/iris/handle/10665/79059

2. GBD 2015 Risk Factors Collaborators. Global, regional, and national comparative risk assessment of 79 behavioural, environmental and occupational, and metabolic risks or clusters of risks, 1990-2015: a systematic analysis for the Global Burden of Disease Study 2015. *Lancet (London, England)* 2016;**388**:1659–724. doi:10.1016/S0140-6736(16)31679-8

For peer review only - http://bmjopen.bmj.com/site/about/guidelines.xhtml

3. World Health Organization. Hypertension. 2019.https://www.who.int/health-topics/hypertension/#tab=tab_1

4. Cuevas AG, Williams DR, Albert MA. Psychosocial Factors and Hypertension: A Review of the Literature. *Cardiol Clin* 2017;**35**:223–30. doi:10.1016/j.ccl.2016.12.004

5. Wilson DK, Kliewer W, Sica DA. The relationship between exposure to violence and blood pressure mechanisms. *Curr Hypertens Rep* 2004;**6**:321–6. doi:10.1007/s11906-004-0028-2

6. Hillis S, Mercy J, Amobi A, *et al.* Global Prevalence of Past-year Violence Against Children: A Systematic Review and Minimum Estimates. *Pediatrics* 2016;**137**:e20154079. doi:10.1542/peds.2015-4079

7. Hughes K, Bellis MA, Hardcastle KA, *et al.* The effect of multiple adverse childhood experiences on health: a systematic review and meta-analysis. *Lancet Public Heal* 2017;**2**:e356–66. doi:10.1016/S2468-2667(17)30118-4

8. Jewkes RK, Dunkle K, Nduna M, *et al.* Associations between childhood adversity and depression, substance abuse and HIV and HSV2 incident infections in rural South African youth. *Child Abuse Negl* 2010;**34**:833–41. doi:10.1016/j.chiabu.2010.05.002

9. Trickett PK, Noll JG, Putnam FW. The impact of sexual abuse on female development: lessons from a multigenerational, longitudinal research study. *Dev Psychopathol* 2011;**23**:453–76. doi:10.1017/S0954579411000174

10. Norman RE, Byambaa M, De R, *et al.* The long-term health consequences of child physical abuse, emotional abuse, and neglect: a systematic review and meta-analysis. *PLoS Med* 2012;**9**:e1001349. doi:10.1371/journal.pmed.1001349

11. Basu A, McLaughlin KA, Misra S, *et al.* Childhood Maltreatment and Health Impact: The Examples of Cardiovascular Disease and Type 2 Diabetes Mellitus in Adults. *Clin Psychol a Publ Div Clin Psychol Am Psychol Assoc* 2017;**24**:125–39. doi:10.1111/cpsp.12191

12. Anderson EL, Fraser A, Caleyachetty R, *et al.* Associations of adversity in childhood and risk factors for cardiovascular disease in mid-adulthood. *Child Abuse Negl* 2018;**76**:138–48. doi:10.1016/j.chiabu.2017.10.015

13. Parrish C, Surkan PJ, Martins SS, *et al.* Childhood adversity and adult onset of hypertension and heart disease in São Paulo, Brazil. *Prev Chronic Dis* 2013;**10**:E205. doi:10.5888/pcd10.130193

14. National Department of Health, Statistics South Africa SAMRC and I. South Africa Demographic and Health Survey 2016. Pretoria, South Africa, and Rockville, Maryland, USA: NDoH, Stats SA, SAMRC, and ICF: 2019.

15. Meinck F, Cluver LD, Boyes ME, *et al.* Physical, emotional and sexual adolescent abuse victimisation in South Africa: prevalence, incidence, perpetrators and locations. *J Epidemiol Community Health* 2016;**70**:910–6. doi:10.1136/jech-2015-205860

16. Gibbs A, Dunkle K, Washington L, *et al.* Childhood traumas as a risk factor for HIV-risk behaviours amongst young women and men living in urban informal settlements in South Africa: A cross-sectional study. *PLoS One* 2018;**13**:e0195369. doi:10.1371/journal.pone.0195369

17. Ward CL, Artz L, Leoschut L, *et al.* Sexual violence against children in South Africa: a nationally representative cross-sectional study of prevalence and correlates. *Lancet Glob Heal* 2018;**6**:e460–8. doi:10.1016/S2214-109X(18)30060-3

For peer review only

For peer review only - http://bmjopen.bmj.com/site/about/guidelines.xhtml

18. Abrahams N, Seedat S, Lombard C, *et al.* Study protocol for a longitudinal study evaluating the impact of rape on women's health and their use of health services in South Africa. *BMJ Open* 2017;**7**:e017296. doi:10.1136/bmjopen-2017-017296

19. Abrahams N, Mhlongo S, Chirwa E, *et al.* Rape survivors in South Africa: analysis of the baseline socio-demographic and health characteristics of a rape cohort. *Glob Health Action* 2020;**13**:1834769. doi:10.1080/16549716.2020.1834769

20. Bernstein DP, Stein JA, Newcomb MD, *et al.* Development and validation of a brief screening version of the Childhood Trauma Questionnaire. *Child Abuse Negl* 2003;**27**:169–90. doi:10.1016/s0145-2134(02)00541-0

21. Kleber RJ. Trauma and Public Mental Health: A Focused Review. *Front psychiatry* 2019;**10**:451. doi:10.3389/fpsyt.2019.00451

22. Zlotnick C, Davidson J, Shea MT, *et al.* Validation of the Davidson Trauma Scale in a sample of survivors of childhood sexual abuse. *J Nerv Ment Dis* 1996;**184**:255–7. doi:10.1097/00005053-199604000-00010

23. Nduna M, Jewkes RK, Dunkle KL, *et al.* Associations between depressive symptoms, sexual behaviour and relationship characteristics: a prospective cohort study of young women and men in the Eastern Cape, South Africa. *J Int AIDS Soc* 2010;**13**:44. doi:10.1186/1758-2652-13-44

24. Gray MJ, Litz BT, Hsu JL, *et al.* Psychometric properties of the life events checklist. *Assessment* 2004;**11**:330–41. doi:10.1177/1073191104269954

25. Alberti KGMM, Eckel RH, Grundy SM, *et al.* Harmonizing the metabolic syndrome: a joint interim statement of the International Diabetes Federation Task Force on Epidemiology and Prevention; National Heart, Lung, and Blood Institute; American Heart Association; World Heart Federation; International . *Circulation* 2009;**120**:1640–5. doi:10.1161/CIRCULATIONAHA.109.192644

26. Nadine Butler. National Guidelines at a glance : hypercholesterolaemia : review. *SA Pharm J* 2009;**76**:22–33.

27. World Health Organization. *Use of Glycated Haemoglobin (HbA1c) in the Diagnosis of Diabetes Mellitus: Abbreviated Report of a WHO Consultation*. Geneva: 2011.

28. Rosseel Y. lavaan: An R Package for Structural Equation Modeling. *J Stat Softw* 2012;**48**:36.

29. Gilbert R, Widom CS, Browne K, *et al.* Burden and consequences of child maltreatment in high-income countries. *Lancet (London, England)* 2009;**373**:68—81. doi:10.1016/s0140-6736(08)61706-7

30. Su S, Wang X, Pollock JS, *et al.* Adverse childhood experiences and blood pressure trajectories from childhood to young adulthood: the Georgia stress and Heart study. *Circulation* 2015;**131**:1674–81. doi:10.1161/CIRCULATIONAHA.114.013104

31. Riley EH, Wright RJ, Jun HJ, *et al.* Hypertension in adult survivors of child abuse: observations from the Nurses' Health Study II. *J Epidemiol Community Health* 2010;**64**:413–8. doi:10.1136/jech.2009.095109

32. Stein DJ, Scott K, Haro Abad JM, *et al.* Early childhood adversity and later hypertension: data from the World Mental Health Survey. *Ann Clin psychiatry Off J Am Acad Clin Psychiatr* 2010;**22**:19–28.

33. Widom CS, Czaja SJ, Bentley T, *et al.* A prospective investigation of physical health outcomes

For peer review only

For peer review only - http://bmjopen.bmj.com/site/about/guidelines.xhtml

in abused and neglected children: new findings from a 30-year follow-up. *Am J Public Health* 2012;**102**:1135–44. doi:10.2105/AJPH.2011.300636

34. Lehman BJ, Taylor SE, Kiefe CI, *et al.* Relationship of early life stress and psychological functioning to blood pressure in the CARDIA study. *Health Psychol* 2009;**28**:338–46. doi:10.1037/a0013785

35. Widom, C. S., & Morris S. Accuracy of adult recollections of childhood victimization, Part 2: Childhood sexual abuse. *Psychol Assess* 1997;**9**:34–46.

For peer review only

For peer review only - http://bmjopen.bmj.com/site/about/guidelines.xhtml

Table 1: Socio-demographic characteristics and cardiovascular disease risk factors presented by hypertension status

Variables	Overall (N=1797)	Hypertension (n=220)	No hypertension (n=1577)	p-value
Age in years, median (P25-P75)	24 (21-29)	26 (23-30)	24 (21-28)	<0.001
Rape exposed	47.4 (45-50)	53.6 (46.8-60.4)	46.4 (39.6-53.2)	0.054
**Years schooling, % (95%CI)**				0.415
1-7 years	2.8 (1.4-4.2)	3.6 (0.4-7.6)	2.7 (1.2-4.1)	
8-12 years	89 (87.6-90.4)	90 (86.8-93.9)	88.8 (87.4-90.3)	
>12 years	8.2 (6.9-9.6)	6.4 (3.2-10.3)	8.5 (7-10)	
**Employment status, % (95%CI)**				
Employed, **% (95%CI)**	20.5 (18.6-22.4)	24.1 (18.6-30.3)	20 (18-22)	0.184
**Residence, % (95%CI)**	**N=1774**	**n=218**	**n=1556**	0.790
Formal urban	72 (70-74)	74 (68.3-79.6)	71.7 (69.5-74)	
Informal urban	16.6 (14.5-18.7)	15 (9.6-20.9)	16.8 (14.6-19)	
Rural/semi-rural	11.4 (9.4-13.6)	11 (5.5-16.7)	11.5 (9.3-13.8)	
**HIV-positive, % (95%CI)**	43.3 (40.9-45.6)	52.7 (45.9-59.5)	42 (39.5-44.4)	0.003
**Lifetime trauma event exposure:**				
Other traumatic exposures score, median (P25-P75)	2 (1-3)	2 (1-4)	2 (1-3)	<0.001
**Health behaviours, % (95%CI)**				
Current smoker	12.5 (11-14)	13.6 (9.4-18.9)	12.3 (10.7-14)	0.651
Current alcohol consumption	53.1 (50.7-55.4)	61.8 (55-68.2)	51.9 (49.4-54.4)	0.006
**Mental health disorders**				
Depression score	21 (11-36)	20 (11-36)	28 (14-38)	<0.001
PTSD score, median (P25-P75)	16 (2-37)	22 (5-41)	15 (1-37)	0.001
**Cardiometabolic health**				
Mean (SD) or Median (P25-P75)				
Systolic blood pressure, mmHg	105 (8.5)	108 (11.2)	104 (7.9)	<0.001
Diastolic blood pressure, mmHg	70 (7.6)	73 (10.3)	70 (7.1)	<0.001
Waist circumference, cm	84 (13.9)	88 (15)	84 (13.7)	<0.001
Body mass index	27 (6.6)	29 (8.3)	27 (6.3)	<0.001
	**N=1614**	**n=169**	**n=1445**	
Total cholesterol, mmol/L	3.8 (0.82)	4.0 (0.84)	3.8 (0.81)	0.052
Low-density lipoprotein cholesterol (LDL-C), mmol/L	2.2 (0.73)	2.3 (0.74)	2.2 (0.72)	0.385
High-density lipoprotein cholesterol (HDL-C), mmol/L	1.2 (0.33)	1.25 (0.37)	1.2 (0.33)	0.128
Triglycerides, mmol/L	0.72 (0.52-0.98)	0.74 (0.55-1.01)	0.72 (0.52-0.98)	0.402
Glycated haemoglobin (HbA1c), %	5.3 (0.6)	5.4 (0.7)	5.3 (0.5)	0.169
Prevalence, % (95%CI)				
Waist circumference ≥80cm	57.6 (55.3-59.9)	64.1 (57.4-70.4)	56.7 (54.3-59.2)	0.046
Body mass index ≥25kg/m^2	54.4 (52.1-56.7)	60.4 (53.7-66.9)	53.6 (51.1-56.1)	0.065
Total cholesterol >5mmol/L	7.5 (6.3-8.9)	8.9 (5.1-14.2)	7.3 (6-8.8)	0.572
LDL-C >3mmol/L	13.5 (11.9-15.3)	16 (10.8-22.4)	13.2 (11.5-15.1)	0.382
HDL-C <1.2mmol/L	53.6 (51.1-56)	49.7 (41.9-57.5)	54 (51.4-56.6)	0.322
Triglycerides >1.5mmol/L	7.2 (6-8.6)	7.1 (3.7-12.1)	7.3 (6-8.7)	0.999
HbA1c ≥5.7%	21.1 (19.2-23.1)	30 (24-36.5)	19.9 (17.9-21.9)	0.001
History of known diabetes	1.4 (0.9-2)	2.7 (1-5.8)	1.2 (0.7-1.9)	0.112
History of known hypertension	11.6 (10.1-13.1)	94.5 (90.7-97.2)	NA	NA

Hypertension defined as follows: systolic blood pressure ≥140mmHg and/or diastolic blood pressure ≥90mmHg or previously diagnosed with hypertension; current smoker: currently smoke any tobacco products such as cigarettes,

For peer review only - http://bmjopen.bmj.com/site/about/guidelines.xhtml

cigars or pipes; current alcohol consumption, consumed at least one drink in an occasion within a month, respectively (one drink is equivalent to one can/bottle of beer, cider, cooler/glass of wine/tot of spirit); severe depression: total scores ≥16 for depressive symptoms during the past week using Centre for Epidemiologic Studies Depression Scale (CES-D score); PTSD, post-traumatic stress disorder, was measures using Davidson Trauma Scale.

For peer review only

Table 2: Description of childhood maltreatment items overall and by hypertension status

Childhood maltreatment items	Overall (N=1797)			Hypertension (n = 220)			No hypertension (n = 1577)			P-value
**Sexual abuse**	n	%	95%CI	n	%	95%CI	n	%	95%CI	
Someone touched my buttocks or genitals or made me touch them when I did not want to	149	8.3	7.0-9.7	26	11.8	7.9-16.8	123	7.8	6.5-9.2	0.058
I had sex with a man who was 5 years older than me	87	4.8	3.9-5.9	20	9.1	5.6-13.7	67	4.2	3.3-5.4	0.003
I had sex with someone because I was threatened or frightened or forced	63	3.5	2.7-4.5	14	6.4	3.5-10.4	49	3.1	2.3-4.1	0.023
I was forced to have sex against my will by a boyfriend	17	0.9	0.6-1.5	7	3.2	1.3-6.4	10	0.6	0.3-1.2	0.002
**Physical abuse**										
I was beaten at home with a belt or stick or whip or something else which was hard	717	39.9	37.6-42.2	102	46.4	39.6-53.2	615	39.0	36.6-41.4	0.043
I was beaten so hard at home that it left a mark or bruise	231	12.9	11.3-14.5	46	20.9	15.7-26.9	185	11.7	10.2-13.4	<0.001
I was beaten or physically punished at school by a teacher or headmaster	225	12.5	11.0-14.1	37	16.8	12.1-22.4	188	11.9	10.4-13.6	0.051
**Emotional abuse**										
I saw or heard my mother being beaten by her husband or boyfriend	223	12.4	10.9-14.1	36	16.4	11.7-21.9	187	11.8	10.3-13.6	0.073
I was told I was lazy or stupid or weak by someone in my family	322	17.9	16.2-19.8	62	28.2	22.3-34.6	260	16.5	14.7-18.4	<0.001
I was insulted or humiliated by someone in my family in front of other people	181	10.1	8.7-11.6	35	15.9	11.3-21.4	146	9.3	7.9-10.8	0.003
**Parental neglect**										
I lived in different households at different times	402	22.4	20.5-24.4	63	28.6	22.8-35.1	339	21.5	19.5-23.6	0.021
Both of my parents were too drunk or drugged to take care of me	43	2.4	1.7-3.2	6	2.7	1-5.8	37	2.3	1.6-3.2	0.641
I spent time outside the home and none of the adults at home knew where I was	108	6.0	4.9-7.2	19	8.6	5.3-13.2	89	5.6	4.6-6.9	0.110

For peer review only - http://bmjopen.bmj.com/site/about/guidelines.xhtml

Table 3: Prevalence of childhood maltreatment types overall and by hypertension status

Number, (%)	Overall (N=1797)			Hypertension (n=220)			No hypertension (n=1577)			P-value
	n	%	95%CI	n	%	95%CI	n	%	95%CI	
**Ever exposed to childhood maltreatment**										
Sexual abuse	241	13.4	11.9-15.1	46	20.9	15.7-26.9	195	12.4	10.8-14.1	<0.001
Physical abuse	768	42.7	40.4-45.1	114	51.8	41.4-54.9	654	41.5	39-43.9	0.004
Emotional abuse	523	29.1	27-31.3	88	40.0	33.4-46.8	435	27.6	25.4-29.9	<0.001
Parental neglect	482	26.8	24.8-28.9	77	35.0	28.7-41.7	405	25.7	23.5-27.9	0.004
Any childhood maltreatment	1058	58.9	56.5-61.2	156	70.9	64.4-76.8	902	57.2	54.7-59.6	<0.001
**Exposure to multiple types of childhood maltreatment**										<0.001
No abuse	739	41.1	38.6-43.6	64	29.1	22.3-36.4	675	42.8	40.1-45.5	
1-2 types	778	43.3	40.8-45.8	102	46.4	39.5-53.7	676	42.9	40.2-45.5	
3-4 types	280	15.6	13.1-18.1	54	24.5	17.7-31.8	226	14.3	11.7-17	
**Combined frequency and severity of childhood maltreatment**										
**Sexual abuse**										<0.001
Never	1556	86.6	85.1-88.1	174	79.1	74.1-84.2	1382	87.6	86.1-89.2	
Some	156	8.7	7.2-10.2	25	11.4	6.4-16.5	131	8.3	6.8-9.9	
Often	85	4.7	3.3-6.3	21	9.5	4.5-14.7	64	4.1	2.5-5.6	
**Physical abuse**										0.002
Never	1029	57.3	54.9-59.7	106	48.2	41.4-55.4	923	58.5	56.1-61.1	
Some	378	21.0	18.7-23.5	48	21.8	15-29	330	20.9	18.5-23.5	
Often	390	21.7	19.4-24.1	66	30.0	23.2-37.2	324	20.5	18.1-23.1	
**Emotional abuse**										<0.001
Never	1274	70.9	68.8-73	132	60.0	53.6-66.8	1142	72.4	70.3-74.6	
Some	290	16.1	14.1-18.3	44	20.0	13.6-26.7	246	15.6	13.4-17.8	
Often	233	13.0	10.9-15.1	44	20.0	13.6-26.7	189	12.0	9.8-14.2	
**Parental neglect**										0.003
Never	1315	73.2	71.2-75.2	143	65.0	59.1-71.6	1172	74.3	72.2-76.5	
Some	328	18.3	16.2-20.3	47	21.4	15.4-28	281	17.8	15.7-20	
Often	154	8.6	6.6-10.6	30	13.6	7.7-20.3	124	7.9	5.8-10	

Frequency and severity of childhood maltreatment defined as: Some: affirmative response of "sometimes" to 1 item only in a specific maltreatment category; Often: affirmative response of "sometime" to >1 item, or response of "often" or "very often" to at least 1 item in a specific maltreatment category.

For peer review only - http://bmjopen.bmj.com/site/about/guidelines.xhtml

Table 4: Logistic regression analyses for the associations of childhood maltreatment with hypertension

Childhood maltreatment (CM) variable	Sample size	HTN, n (%)	Unadjusted Odds Ratio	P-value	P-interaction (CM*RE)	Adjusted Odds Ratio	P-value
**Ever exposed to CM**							
Any CM					0.083		
No	739	64 (8.7)	1.00			1.00	
Yes	1058	156 (14.7)	1.82 (1.35-2.50)	<0.001		1.62 (1.19-2.25)	0.002
Sexual abuse					0.081		
No	1556	174 (11.2)	1.00			1.00	
Yes	241	46 (19.1)	1.87 (1.30-2.66)	<0.001		1.64 (1.12-2.37)	0.009
Physical abuse					0.468		
No	1029	106 (10.3)	1.00			1.00	
Yes	768	114 (14.8)	1.52 (1.14-2.02)	0.004		1.43 (1.07-1.92)	0.016
Emotional abuse					0.201		
No	1274	132 (10.4)	1.00			1.00	
Yes	523	88 (16.8)	1.75 (1.30-2.34)	<0.001		1.57 (1.16-2.13)	0.003
Parental neglect					0.557		
No	1315	143 (10.9)	1.00			1.00	
Yes	482	77 (16.0)	1.56 (1.15-2.09)	0.004		1.37 (1.00-1.86)	0.049
**Exposure to multiple types of CM**					0.101		
No abuse	739	64 (8.7)	1.00			1.00	
1-2 abuse types	778	102 (13.1)	1.59 (1.15-2.22)	0.005		1.41 (1.01-1.99)	0.046
3-4 abuse types	280	54 (19.3)	2.52 (1.70-3.72)	<0.001		1.81 (1.18-2.76)	0.006
**Frequency and severity of CM**							
Cumulative maltreatment (range 0-8)	1797	220 (12.2)	1.19 (1.12-1.28)	<0.001	0.061	1.13 (1.05-1.21)	0.002
Sexual abuse					0.081		
Never	1556	174 (11.2)	1.00			1.00	
Some	156	25 (16.0)	1.52 (0.94-2.35)	0.073		1.39 (0.86-2.19)	0.164
Often	85	21(24.7)	2.61 (1.52-4.30)	<0.001		2.12 (1.21-3.60)	0.007
Physical abuse					0.378		
Never	1029	106 (10.3)	1.00			1.00	
Some	378	48 (12.7)	1.27 (0.87-1.81)	0.202		1.24 (0.85-1.79)	0.246
Often	390	66 (16.9)	1.77 (1.27-2.47)	<0.001		1.62 (1.14-2.27)	0.006
Emotional abuse					0.116		
Never	1274	132 (10.4)	1.00			1.00	
Some	290	44 (15.2)	1.55 (1.06-2.22)	0.020		1.44 (0.97-2.08)	0.061
Often	233	44 (18.9)	2.01 (1.37-2.91)	<0.001		1.75 (1.17-2.57)	0.005
Parental neglect					0.139		
Never	1315	143 (10.9)	1.00			1.00	
Some	328	47 (14.3)	1.37 (0.95-1.94)	0.081		1.21 (0.84-1.73)	0.302
Often	154	30 (19.5)	1.98 (1.26-3.03)	0.002		1.74 (1.09-2.71)	0.016

CM, childhood maltreatment; RE, rape exposure; logistic regression models adjusted for age, BMI, recent rape exposure, current smoking; current alcohol use, HIV infection, dysglycemia (HbA1c≥5.7% and or previously diagnosed with diabetes),

For peer review only

For peer review only - http://bmjopen.bmj.com/site/about/guidelines.xhtml

other traumatic exposures scores, depression scores and post-traumatic stress disorder (PTSD) scores. Current smoking: currently smoke any tobacco products such as cigarettes, cigars or pipes; current alcohol consumption: consumed at least one drink in an occasion within a month, respectively (one drink is equivalent to one can/bottle of beer, cider, cooler/glass of wine/tot of spirit); depression scores, depressive symptoms during the past week using Centre for Epidemiologic Studies Depression Scale (CES-D score); PTSD (scores), post-traumatic stress disorder measured using Davidson Trauma Scale, a validated self-rating scale. Cumulative maltreatment: ranged from 0 (no maltreated in any type) to 8 (often/severely maltreated in all 4 types).

For peer review only

For peer review only - http://bmjopen.bmj.com/site/about/guidelines.xhtml

Table 5: Results of mediation analysis for the effects of childhood maltreatment on adult prevalent hypertension in a sample of South African women (N=1797)

Confounder/ mediator	Total effect			Direct effect			Indirect effect			Proportions of mediation effects
	Estimates	95% CI	P-value	Estimates	95% CI	P-value	Estimates	95% CI	P-value	% (95%CI)
**Any CM**	0.317	0.160-0.487	<0.001							
BMI				0.310	0.155-0.467	<0.001	0.007	-0.005-0.003	0.836	3.3 (-1.7-10.4)
HbA1c				0.318	0.165-0.479	<0.001	-0.001	-0.016-0.011	0.899	-1.6 (-4.2-2.4)
HIV-positive				0.314	0.154-0.477	<0.001	0.004	-0.014-0.024	0.655	0.9 (-3.3-6.8)
Rape exposure				0.301	0.142-0.470	<0.001	0.015	-0.001-0.038	0.147	3.5 (0.3-11.5)
Current smoking				0.311	0.154-0.480	<0.001	0.004	-0.033-0.040	0.826	0.5 (-3.8-6.8)
Current alcohol				0.278	0.116-0.452	<0.001	0.038	0.007-0.079	0.036	8.2 (1.3-22)
Depressive symptom score				0.280	0.123-0.446	<0.001	0.037	0.012-0.066	0.007	13 (4.4-31)
PTSD score				0.283	0.130-0.445	<0.001	0.034	0.006-0.064	0.024	11.5 (1.9-29)
Other traumatic exposures score				0.232	0.073-0.403	0.006	0.089	0.052-0.127	<0.001	32.9 (16.6-68.2)
**Multiple CM types**	0.249	0.144-0.358	<0.001							
BMI				0.244	0.140-0.351	<0.001	0.005	-0.004-0.016	0.363	1.6 (-1.9-7.3)
HbA1c				0.249	0.142-0.360	<0.001	0.000	-0.013-0.011	0.948	0.1 (-2-3.5)
HIV-positive				0.241	0.137-0.348	<0.001	0.007	-0.004-0.055	0.283	1.6 (-0.6-6.8)
Rape exposure				0.236	0.127-0.343	<0.001	0.012	-0.002-0.029	0.144	3 (0.1-9.4)
Current smoking				0.245	0.138-0.359	<0.001	0.002	-0.029-0.032	0.916	0.3 (-5.6-5.2)

For peer review only - http://bmjopen.bmj.com/site/about/guidelines.xhtml

Current alcohol				0.221	0.114-0.330	<0.001	0.027	0.003-0.055	0.043	6.4 (0.5-15.6)
Depressive symptom score				0.241	0.114-0.330	<0.001	0.029	0.008-0.053	0.011	12 (3.5-26)
PTSD score				0.223	0.117 0.331	<0.001	0.024	0.001-0.048	0.045	10.2 (1.7-25.3)
Other traumatic exposures score				0.179	0.069-0.292	0.001	0.072	0.041-0.104	<0.001	30.9 (16-59)
**Cumulative CM score**	0.096	0.058-0.135	<0.001							
BMI				0.096	0.058-0.134	<0.001	0.000	-0.003-0.004	0.829	0.3 (-3.6-5.2)
HbA1c				0.097	0.059-0.135	<0.001	-0.001	-0.006 – 0.003	0.500	-0.4 (-4.2-1.5)
HIV-positive				0.095	0.056-0.133	<0.001	0.002	-0.003-0.007	0.422	1.1 (-1.2-4.8)
Rape exposure				0.090	0.052-0.129	<0.001	0.005	-0.002-0.013	0.152	3.5 (-0.1-10.2)
Current smoking				0.095	0.056-0.134	<0.001	0.000	-0.010 – 0.011	0.955	-0.4 (-5.1-4.7)
Current alcohol				0.087	0.048-0.126	<0.001	0.009	0.001-0.018	0.038	5.2 (0.5-12.8)
Depressive symptom score				0.084	0.045-0.123	<0.001	0.012	0.002 – 0.022	0.022	11.6 (2.9-24.9)
PTSD score				0.086	0.047-0.126	<0.001	0.009	0.000-0.020	0.066	9.6 (1.2-24.9)
Other traumatic exposures score				0.067	0.027-0.107	0.001	0.030	0.017-0.042	<0.001	30.8 (15.6-58.6)

Data are standardized beta co-efficient. CM, childhood maltreatment; current smoking: currently smoke any tobacco products such as cigarettes, cigars or pipes; current alcohol consumption: consumed at least one drink in an occasion within a month, respectively (one drink is equivalent to one can/bottle of beer, cider, cooler/glass of wine/tot of spirit); depressive symptom (scores): depressive symptoms during the past week using Centre for Epidemiologic Studies Depression Scale (CES-D score); PTSD (scores): post-traumatic stress disorder measured using Davidson Trauma Scale, a validated self-rating scale. Cumulative abuse (score): ranged from 0 (no abused in any type) to 8 (often/severely abused in all 4 types).

For peer review only

For peer review only - http://bmjopen.bmj.com/site/about/guidelines.xhtml

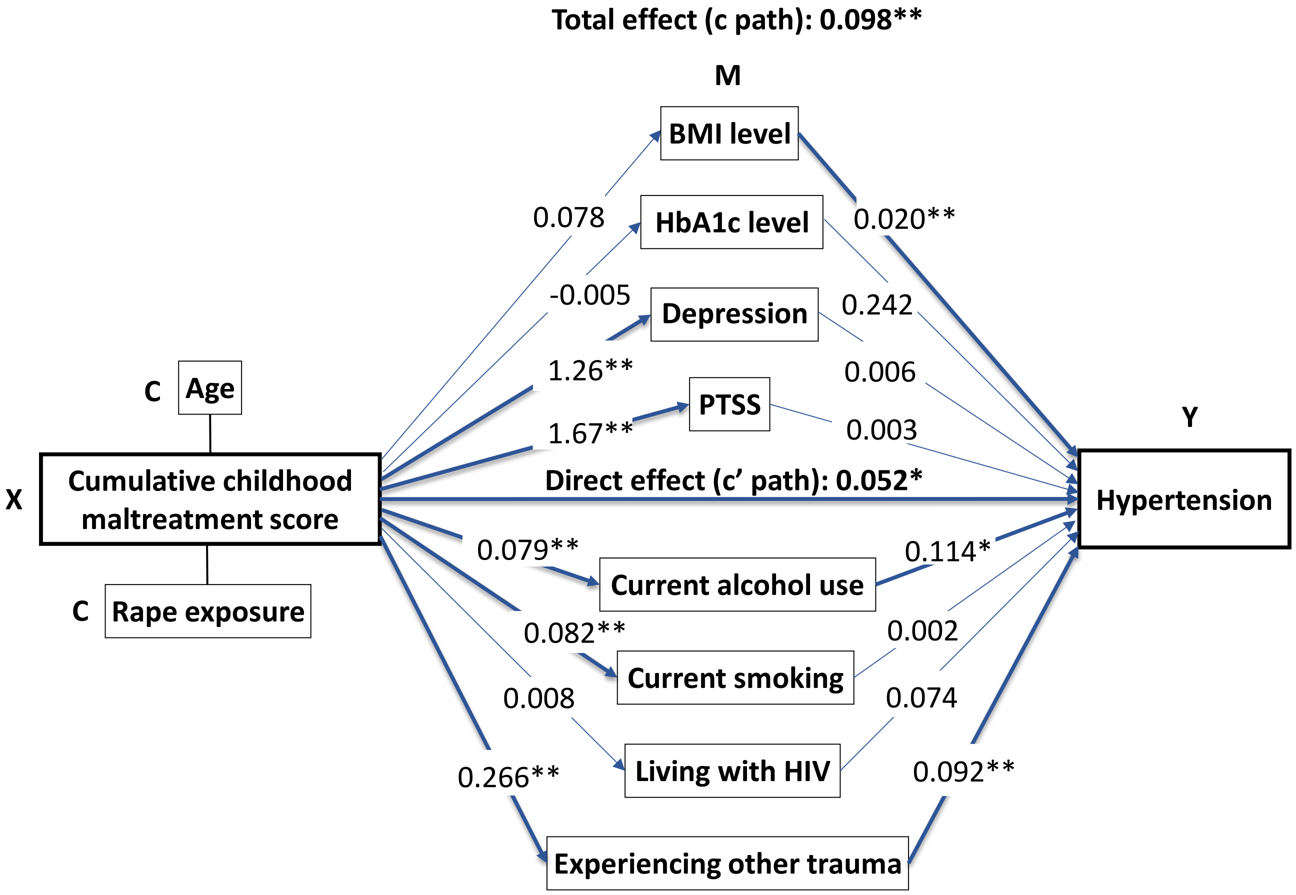

Figure 1: Multiple mediation model of the association of cumulative childhood maltreatment score with adult prevalent hypertension, adjusted for age and rape exposure. All path estimates are standardized regression coefficients. * p<0.05, ** p<0.001. PTSS: post-traumatic stress symptoms.

For peer review only - http://bmjopen.bmj.com/site/about/guidelines.xhtml

Table S1: Results of mediation analysis for the effects of childhood maltreatment on adult prevalent hypertension in a sample of South African women (N=1797)

Independent variables	Total effect			Direct effect			Indirect effect		
	Estimates	95% CI	P-value	Estimates	95% CI	P-value	Estimates	95% CI	P-value
**Any CM**	1.78	1.34-2.43	<0.001						
BMI				1.76	1.33-2.34	<0.001	1.01	0.99-1.01	0.836
HbA1c				1.78	1.35-2.39	<0.001	0.99	0.97-1.02	0.899
HIV-positive				1.77	1.32-2.38	<0.001	1.01	0.97-1.04	0.655
Rape exposure				1.73	1.29-2.35	<0.001	1.03	0.99-1.07	0.147
Current smoking				1.76	1.32-2.40	<0.001	1.01	0.94-1.08	0.826
Current alcohol				1.66	1.24-2.78	<0.001	1.07	1.01-1.15	0.036
Depressive symptom score				1.66	1.25-2.25	<0.001	1.07	1.02-1.13	0.007
PTSD score				1.67	1.27-2.25	<0.001	1.06	1.01-1.12	0.024
Other traumatic exposures score				1.53	1.14-2.08	0.006	1.18	1.10-1.26	<0.001
**Multiple CM types**	1.58	1.30-1.92	<0.001						
BMI				1.56	1.29-1.89	<0.001	1.01	0.99-1.03	0.363
HbA1c				1.57	1.29-1.93	<0.001	1.00	0.97-1.02	0.948
HIV-positive				1.55	1.28-1.88	<0.001	1.01	0.99-1.10	0.283
Rape exposure				1.54	1.26-1.87	<0.001	1.02	1.00-1.05	0.144
Current smoking				1.56	1.29-1.92	<0.001	1.00	0.95-1.06	0.916
Current alcohol				1.50	1.23-1.82	<0.001	1.05	1.01-1.11	0.043
Depressive symptom score				1.55	1.23-1.82	<0.001	1.05	1.01-1.10	0.011
PTSD score				1.50	1.24-1.83	<0.001	1.04	1.01-1.09	0.045
Other traumatic exposures score				1.39	1.13-1.70	0.001	1.14	1.08-1.21	<0.001
**Cumulative CM score**	1.19	1.11-1.28	<0.001						
BMI				1.19	1.11-1.28	<0.001	1.00	0.99-1.01	0.829
HbA1c				1.19	1.11-1.28	<0.001	1.00	0.99-1.01	0.500
HIV-positive				1.19	1.11-1.27	<0.001	1.00	0.99-1.01	0.422
Rape exposure				1.18	1.10-1.26	<0.001	1.01	0.99-1.02	0.152
Current smoking				1.19	1.11-1.28	<0.001	1.00	0.98-1.02	0.955
Current alcohol				1.17	1.09-1.26	<0.001	1.02	1.01-1.03	0.038
Depressive symptom score				1.17	1.09-1.25	<0.001	1.02	1.01-1.04	0.022
PTSD score				1.17	1.09-1.26	<0.001	1.02	1.00-1.04	0.066
Other traumatic exposures score				1.13	1.05-1.21	0.001	1.06	1.03-1.08	<0.001

For peer review only

Data are odds ratios from probit models. CM, childhood maltreatment; current smoking: currently smoke any tobacco products such as cigarettes, cigars or pipes; current alcohol consumption: consumed at least one drink in an occasion within a month, respectively (one drink is equivalent to one can/bottle of beer, cider, cooler/glass of wine/tot of spirit); depressive symptom (scores): depressive symptoms during the past week using Centre for Epidemiologic Studies Depression Scale (CES-D score); PTSD (scores): post-traumatic stress disorder measured using Davidson Trauma Scale, a validated self-rating scale. Cumulative abuse (score): ranged from 0 (no abused in any type) to 8 (often/severely abused in all 4 types).

Table S2: Results of multiple mediation analysis for the effects of childhood maltreatment on adult prevalent hypertension adjusted for age and rape exposure in a sample of South African women (N=1797)

Independent variables	Total effect			Direct effect			Indirect effect		
	Estimates	95% CI	P-value	Estimates	95% CI	P-value	Estimates	95% CI	P-value
**Any CM**	0.335	0.166-0.505	<0.001	0.163	-0.022-0.347	0.084			
BMI							0.010	-0.004-0.023	0.154
HbA1c							-0.002	-0.015-0.011	0.768
HIV-positive							0.002	-0.008-0.011	0.694
Current smoking							0.003	-0.030-0.035	0.867
Current alcohol							0.038	0.003-0.073	0.034
Depressive symptom score							0.026	0.001-0.051	0.040
PTSS score							0.020	-0.013-0.053	0.232
Other traumatic exposures score							0.076	0.040-0.113	<0.001
**Multiple CM types**									
BMI	0.256	0.146-0.367	<0.001	0.127	0.003-0.251	0.045	0.007	-0.003-0.016	0.160
HbA1c							-0.001	-0.009-0.007	0.872
HIV-positive							0.003	-0.004-0.011	0.414
Current smoking							0.001	-0.026-0.028	0.947
Current alcohol							0.027	0.002-0.052	0.040
Depressive symptom score							0.020	-0.001-0.041	0.067
PTSD score							0.012	-0.013-0.038	0.337
Other traumatic exposures score							0.061	0.032-0.090	<0.001
**Cumulative CM score**									
BMI	0.098	0.059-0.138	<0.001	0.052	0.007-0.097	0.024	0.002	-0.002-0.005	0.337
HbA1c							-0.001	-0.004 – 0.002	0.364
HIV-positive							0.001	-0.002-0.003	0.655
Current smoking							0.000	-0.010 – 0.010	0.976

For peer review only

Current alcohol							0.009	0.001-0.018	0.038
Depressive symptom score							0.007	-0.001 – 0.016	0.090
PTSD score							0.004	-0.006-0.015	0.425
Other traumatic exposures score							0.024	0.012-0.037	<0.001

Data are standardized co-efficient. CM, childhood maltreatment; current smoking: currently smoke any tobacco products such as cigarettes, cigars or pipes; current alcohol consumption: consumed at least one drink in an occasion within a month, respectively (one drink is equivalent to one can/bottle of beer, cider, cooler/glass of wine/tot of spirit); depressive symptom (scores): depressive symptoms during the past week using Centre for Epidemiologic Studies Depression Scale (CES-D score); PTSD (scores): post-traumatic stress disorder measured using Davidson Trauma Scale, a validated self-rating scale. Cumulative abuse (score): ranged from 0 (no abused in any type) to 8 (often/severely abused in all 4 types).

Table S3: Results of multiple mediation analysis for the effects of childhood maltreatment on adult prevalent hypertension adjusted for age and rape exposure in a sample of South African women (N=1797)

**Independent variables**	**Total effect**			**Direct effect**			**Indirect effect**		
	Estimates	95% CI	P-value	Estimates	95% CI	P-value	Estimates	95% CI	P-value
**Any CM**	1.84	1.35-2.51	<0.001	1.35	0.96-1.88	0.084			
BMI							1.02	0.99-1.04	0.154
HbA1c							0.99	0.97-1.02	0.768
HIV-positive							1.00	0.98-1.02	0.694
Current smoking							1.01	0.95-1.07	0.867
Current alcohol							1.07	1.01-1.14	0.034
Depressive symptom score							1.05	1.01-1.10	0.040
PTSS score							1.04	0.98-1.10	0.232
Other traumatic exposures score							1.14	1.07-1.23	<0.001
**Multiple CM types**	1.59	1.30-1.95	<0.001	1.26	1.01-1.58	0.045			
BMI							1.01	0.99-1.03	0.160
HbA1c							1.00	0.98-1.01	0.872
HIV-positive							1.01	0.99-1.02	0.414
Current smoking							1.00	0.95-1.05	0.947
Current alcohol							1.05	1.01-1.10	0.040
Depressive symptom score							1.04	1.00-1.08	0.067
PTSD score							1.02	0.98-1.07	0.337
Other traumatic exposures score							1.12	1.06-1.18	<0.001

**Cumulative CM score**	1.19	1.11-1.29	<0.001	1.10	1.01-1.19	0.024			
BMI							1.00	0.99-1.01	0.337
HbA1c							1.00	0.99-1.01	0.364
HIV-positive							1.00	0.99-1.01	0.655
Current smoking							1.00	0.98-1.02	0.976
Current alcohol							1.02	1.01-1.03	0.038
Depressive symptom score							1.01	1.00-1.03	0.090
PTSD score							1.01	0.99-1.03	0.425
Other traumatic exposures score							1.04	1.02-1.07	<0.001

Data are odds ratios from probit model. CM, childhood maltreatment; current smoking: currently smoke any tobacco products such as cigarettes, cigars or pipes; current alcohol consumption: consumed at least one drink in an occasion within a month, respectively (one drink is equivalent to one can/bottle of beer, cider, cooler/glass of wine/tot of spirit); depressive symptom (scores): depressive symptoms during the past week using Centre for Epidemiologic Studies Depression Scale (CES-D score); PTSD (scores): post-traumatic stress disorder measured using Davidson Trauma Scale, a validated self-rating scale. Cumulative abuse (score): ranged from 0 (no abused in any type) to 8 (often/severely abused in all 4 types).

For peer review only

STROBE Statement—Checklist of items that should be included in reports of *cross-sectional studies*

	Item No	Recommendation	Page No
**Title and abstract**	1	(*a*) Indicate the study's design with a commonly used term in the title or the abstract	1-2
		(*b*) Provide in the abstract an informative and balanced summary of what was done and what was found	2
**Introduction**			
Background/rationale	2	Explain the scientific background and rationale for the investigation being reported	3-4
Objectives	3	State specific objectives, including any prespecified hypotheses	4
**Methods**			
Study design	4	Present key elements of study design early in the paper	4
Setting	5	Describe the setting, locations, and relevant dates, including periods of recruitment, exposure, follow-up, and data collection	4-5
Participants	6	(*a*) Give the eligibility criteria, and the sources and methods of selection of participants	4-5
Variables	7	Clearly define all outcomes, exposures, predictors, potential confounders, and effect modifiers. Give diagnostic criteria, if applicable	5-7
Data sources/ measurement	8*	For each variable of interest, give sources of data and details of methods of assessment (measurement). Describe comparability of assessment methods if there is more than one group	5-7
Bias	9	Describe any efforts to address potential sources of bias	NA
Study size	10	Explain how the study size was arrived at	4
Quantitative variables	11	Explain how quantitative variables were handled in the analyses. If applicable, describe which groupings were chosen and why	7-8
Statistical methods	12	(*a*) Describe all statistical methods, including those used to control for confounding	7-8
		(*b*) Describe any methods used to examine subgroups and interactions	7-8
		(*c*) Explain how missing data were addressed	NA
		(*d*) If applicable, describe analytical methods taking account of sampling strategy	7-8
		(*e*) Describe any sensitivity analyses	NA
**Results**			
Participants	13*	(a) Report numbers of individuals at each stage of study—eg numbers potentially eligible, examined for eligibility, confirmed eligible, included in the study, completing follow-up, and analysed	9
		(b) Give reasons for non-participation at each stage	NA
		(c) Consider use of a flow diagram	NA
Descriptive data	14*	(a) Give characteristics of study participants (eg demographic, clinical, social) and information on exposures and potential confounders	10
		(b) Indicate number of participants with missing data for each variable of interest	9-10
Outcome data	15*	Report numbers of outcome events or summary measures	10-11

For peer review only - http://bmjopen.bmj.com/site/about/guidelines.xhtml

Main results	16	(*a*) Give unadjusted estimates and, if applicable, confounder-adjusted estimates and their precision (eg, 95% confidence interval). Make clear which confounders were adjusted for and why they were included	10-11
		(*b*) Report category boundaries when continuous variables were categorized	10-11
		(*c*) If relevant, consider translating estimates of relative risk into absolute risk for a meaningful time period	NA
Other analyses	17	Report other analyses done—eg analyses of subgroups and interactions, and sensitivity analyses	NA
**Discussion**			
Key results	18	Summarise key results with reference to study objectives	11
Limitations	19	Discuss limitations of the study, taking into account sources of potential bias or imprecision. Discuss both direction and magnitude of any potential bias	13-14
Interpretation	20	Give a cautious overall interpretation of results considering objectives, limitations, multiplicity of analyses, results from similar studies, and other relevant evidence	12-14
Generalisability	21	Discuss the generalisability (external validity) of the study results	12-15
**Other information**			
Funding	22	Give the source of funding and the role of the funders for the present study and, if applicable, for the original study on which the present article is based	15

*Give information separately for exposed and unexposed groups.

Note: An Explanation and Elaboration article discusses each checklist item and gives methodological background and published examples of transparent reporting. The STROBE checklist is best used in conjunction with this article (freely available on the Web sites of PLoS Medicine at http://www.plosmedicine.org/, Annals of Internal Medicine at http://www.annals.org/, and Epidemiology at http://www.epidem.com/). Information on the STROBE Initiative is available at www.strobe-statement.org.

For peer review only

The associations of childhood maltreatment with hypertension in South African women: A cross-sectional study

Journal:	*BMJ Open*
Manuscript ID	bmjopen-2021-057436.R1
Article Type:	Original research
Date Submitted by the Author:	05-Jul-2022
Complete List of Authors:	NGUYEN, KIM ANH; South African Medical Research Council, Non-Communicable Diseases Research Unit<br>Kengne , AP; South African Medical Research Council, Non-Communicable Diseases Research Unit; University of Cape Town Department of Medicine<br>Abrahams, Naeemah; South African Medical Research Council, Gender and Health Research Unit<br>Jewkes, Rachel; South African Medical Research Council, Gender and Health Research Unit; South African Medical Research Council, Office of the Executive Scientist<br>Mhlongo, Shibe; South African Medical Research Council, Gender & Health Research Unit<br>Peer, Nasheeta; South African Medical Research Council, Non-communicable Diseases Research Unit; University of Cape Town Department of Medicine
**Primary Subject Heading**:	Public health
Secondary Subject Heading:	Mental health
Keywords:	Hypertension < CARDIOLOGY, PUBLIC HEALTH, EPIDEMIOLOGY, MENTAL HEALTH, Child protection < PAEDIATRICS

I, the Submitting Author has the right to grant and does grant on behalf of all authors of the Work (as defined in the below author licence), an exclusive licence and/or a non-exclusive licence for contributions from authors who are: i) UK Crown employees; ii) where BMJ has agreed a CC-BY licence shall apply, and/or iii) in accordance with the terms applicable for US Federal Government officers or employees acting as part of their official duties; on a worldwide, perpetual, irrevocable, royalty-free basis to BMJ Publishing Group Ltd ("BMJ") its licensees and where the relevant Journal is co-owned by BMJ to the co-owners of the Journal, to publish the Work in this journal and any other BMJ products and to exploit all rights, as set out in our licence.

The Submitting Author accepts and understands that any supply made under these terms is made by BMJ to the Submitting Author unless you are acting as an employee on behalf of your employer or a postgraduate student of an affiliated institution which is paying any applicable article publishing charge ("APC") for Open Access articles. Where the Submitting Author wishes to make the Work available on an Open Access basis (and intends to pay the relevant APC), the terms of reuse of such Open Access shall be governed by a Creative Commons licence – details of these licences and which Creative Commons licence will apply to this Work are set out in our licence referred to above.

Other than as permitted in any relevant BMJ Author's Self Archiving Policies, I confirm this Work has not been accepted for publication elsewhere, is not being considered for publication elsewhere and does not duplicate material already published. I confirm all authors consent to publication of this Work and authorise the granting of this licence.

For peer review only

The associations of childhood maltreatment with hypertension in South African women: A cross-sectional study

Kim Anh Nguyen [1], Andre Pascal Kengne [1,2], Naeemah Abrahams [3], Rachel Jewkes [3,4], Shibe Mhlongo [3], Nasheeta Peer [1,2]

[1] Non-communicable Diseases Research Unit, South African Medical Research Council, Cape Town and Durban, South Africa

[2] Department of Medicine, University of Cape Town, Cape Town, South Africa

[3] Gender and Health Research Unit, South African Medical Research Council, Cape Town and Pretoria, South Africa

[4] Office of the Executive Scientist, South African Medical Research Council, Cape Town, South Africa

Corresponding author:

Dr Kim Anh Nguyen

Postal address: Non-communicable Diseases Research Unit, South African Medical Research Council, PO Box 19070 Tygerberg, 7505 Cape Town, South Africa

Email: Kim.Nguyen@mrc.ac.za

Abstract word count # 297

Main text word count # 4833

Number of main Tables # 5

Number of Supplement Tables # 4

Number of Figures # 1

Number of Supplement Figures # 2

Number of References # 39

For peer review only

For peer review only - http://bmjopen.bmj.com/site/about/guidelines.xhtml

ABSTRACT

Objectives: To examine the associations of childhood maltreatment (CM) with hypertension, and the mediating effects of hypertension risk factors on the associations in South African women, using baseline data of the Rape Impact Cohort Evaluation (RICE) longitudinal study.

Design: Cross-sectional

Setting and participants: Self-reported data on CM exposure and its severity in 18-40-year-old women living in KwaZulu-Natal province were assessed. Logistic regression models, adjusted for traditional hypertension risk factors, rape exposure, HIV-infection, other traumatic exposures, depression scores and acute stress reactions (ASR) scores, were used to examine the CM-hypertension associations.

Results: Among 1797 women, 220 (12.2%) had hypertension; the CM prevalence was marginally higher in women with hypertension than without hypertension overall (70.9% vs 57.2%) and for each abuse type: sexual abuse (20.9% vs 12.4%), physical abuse (51.8% vs 41.5%), emotional abuse (40% vs 27.6%) and parental neglect (35% vs 25.7%). Exposures to 1-2 types and 3-4 types of CM were 46.4% and 24.5%, respectively, in women with hypertension, and lower in women without (42.9% and 14.3%, respectively). Exposures to any CM (adjusted odds ratio: 1.62; 95%CI: 1.19-2.25), sexual abuse (1.64; 1.12-2.37), emotional abuse (1.57; 1.16-2.13), physical abuse (1.43; 1.07-1.92) and parental neglect (1.37; 1.00-1.86) were associated with hypertension. Exposures to an increasing number of abuse types and cumulative severity of CM overall (1.13; 1.05-1.21) and for each CM type were associated with increased odds of hypertension. Alcohol use, other trauma experienced, depression and ASR partially mediated these associations.

Conclusion: CM was associated with hypertension; the effects were greater with multiple abuse types and severe abuse, and were partially mediated by alcohol use, depression, ASR and other traumatic exposures. While CM must be prevented, effective mental health interventions to curb the uptake of unhealthy behaviours and the development of hypertension in women exposed to CM are key.

Key words: hypertension, childhood abuse, childhood maltreatment, sexual abuse, emotional abuse, physical abuse, parental neglect, depression, acute stress reactions, post-traumatic stress disorder, alcohol, stress

For peer review only - http://bmjopen.bmj.com/site/about/guidelines.xhtml

ARTICLE SUMMARY

Strengths and limitations of this study

- This is among few studies from low- and middle-income countries to examine the association between childhood maltreatment and hypertension.
- Blood pressures of the study participants were objectively measured during data collection, unlike other relevant studies that used a self-reported measure of hypertension only.
- Four categories of childhood maltreatment, and their frequency and severity, were assessed, which provides a comprehensive understanding of the relationship of childhood maltreatment with hypertension.
- Retrospective self-report of childhood maltreatment experiences could be subject to under reporting.
- Data were collected only in women and might not be applicable to men.

INTRODUCTION

Hypertension is highly prevalent globally with the greatest burden in sub-Saharan Africa where, at 46%, it is a major contributor to cardiovascular disease morbidity and mortality.[1,2] Together with age, obesity, unhealthy diets, physical inactivity, smoking and alcohol use, psychosocial stressors are among the established drivers of hypertension.[3] Emerging studies have identified exposures to trauma and violence as psychosocial stressors that influence hypertension risk.[4] Childhood traumatic experiences i.e. child abuse is an example of early trauma which may subsequently contribute to the development of hypertension.[5]

Childhood maltreatment (CM) such as sexual abuse, physical abuse, emotional abuse and parental neglect have increasingly been reported in many parts of the world where at least half of the children were exposed to these traumas.[6] The overall prevalence estimated ranges from 12% reported in Europe, to 50% in Africa and 64% in Asia. The impact of CM is well-established with CM-induced traumatic stress contributing to depression, anxiety, post-traumatic stress and maladaptive risky lifestyle behaviours in later life .[7–9] Growing evidence suggests that CM adversely impacts physical health, resulting in a wide range of disorders including cardiovascular disease risk factors such as hypertension. [7,10,11] It is suggested that exposures to CM may independently activate the hypothalamic-pituitary-adrenal axis and the

For peer review only - http://bmjopen.bmj.com/site/about/guidelines.xhtml

sympathetic nervous system and promote inflammation, similar to the postulated pathways for other stressors.[4] These physiological changes increase the production of hormones such as catecholamine and glucocorticoid which raise BP levels. The repeated activation of these systems with multiple or frequent exposures to CM may lead to sustained increases in BP and contribute to the development of hypertension.[4]

Although extensive research has found that chronic psychosocial strain contributes to the development of hypertension overtime,[4] and CM has been considered such a stressor,[5] the current evidence on this relationship is inconsistent. Some studies have demonstrated that CM, particularly sexual abuse and physical abuse, were associated with hypertension.[11] Other studies, however, have shown no such association[12] or only an indirect association via mental health disorders or through the link with obesity.[11] However, these findings were from a few studies conducted mostly in developed countries, with variations in methodologies especially in measurements and definitions of CM as well as the scope of CM investigated.[11–13]

South Africa has high burdens of both hypertension[14] and CM,[15–17] calling for the exploration of an association, if any, between these two factors. Therefore, this study aimed to examine the associations of CM including sexual abuse, physical abuse, emotional abuse and parental neglect with the presence of hypertension in 18-40-year-old South African women, using the baseline data of the Rape Impact Cohort Evaluation (RICE) longitudinal study.[18] Additionally, we explored the mediating effects of potential hypertension risk factors such as body mass index (BMI), glycated haemoglobin (HbA1c), HIV-infection, rape exposure, current smoking, current alcohol consumption, current depressive symptom, acute stress reactions (ASR) and other traumatic exposure on the associations.

METHODS

Study design and population

The RICE cohort study was conducted in the Greater Durban Metro area in the KwaZulu-Natal province from October 2014 to March 2020. The study included women who reported being raped (rape exposed) (n=947) recruited from five public health-based post-rape care service and women who have not ever experienced rape or forced sex (non-rape exposed) (n=852) (controls) recruited from adjacent public health primary healthcare clinics. Both groups of

For peer review only - http://bmjopen.bmj.com/site/about/guidelines.xhtml

women were likely to be similar because they were recruited from the same geographical area and were of similar ages to ensure comparability.[18,19] The details of the study protocol has been published previously.[18] Eligible participants for both arms were women aged 18-40 years while those who were more than 14 weeks pregnant, lactating, had cognitive disabilities or severely mentally distressed rape survivors were excluded. Further exclusion among women in the control arm were those who reported lifetime exposure to rape or force sex. Furthermore, the baseline interviews were done within 20 days of the incident rape event among the rape-exposed participants. This timing of the baseline interview was critical for the primary aim of the main study which was to determine the incidence of HIV infection post rape and therefore the baseline documentation of HIV infection and ASR was critical.

Of note, while the RICE study had two arms, in an attempt to achieve the desired aims, the present study used the data from the pooled group of women, which are described in more detail in the statistical analysis.

Data collection

An electronic data capture system (Bryant) was used for data collection. Trained fieldworkers administered questionnaires, clinical examinations and biochemical assessments. Interview data collected included socio-demographic variables (age, education level, employment status and residence type and area), medical history, lifestyle behaviours (alcohol and tobacco use), exposure to the different childhood exposure types and mental health assessments (depressive symptoms, post-traumatic stress disorder).

Measurements and definitions

Socio-demographic and lifestyle characteristics:

Education level was categorized as primary (1-7 school years), high-school (8-12 school years) and higher; while residence was classified as formal urban, informal urban, rural and semi-rural. Current alcohol consumption was defined as consumed at least one drink within a month (one drink equivalent to one can/bottle of beer, cider, cooler/glass of wine/tot of spirit); current smoking as “currently smoke any tobacco products such as cigarettes, cigars or pipes”.

For peer review only - http://bmjopen.bmj.com/site/about/guidelines.xhtml

Childhood maltreatment and mental health outcomes:

CM was assessed using the Childhood Trauma Questionnaire Short Form (CTQ-SF) scale which was adapted and used previously in South Africa.[8,20] Thirteen items were used to measure four abuse types: sexual abuse (4 items), emotional abuse (3 items), physical abuse (3 items), and parental neglect (3 items). Participants' exposure to each of these items before 18 years of age was determined. Exposures were evaluated as yes/no for each type of abuse followed by the frequency of exposure (never (score 0), some (score 1), often (score 2)). Overall CM was defined as exposure to any of the four abuse types and categorized as no exposure (coded 0) and exposure to any abuse (coded 1). Exposure to multiple combined CM types was coded as none (coded 0), 1-2 types (coded 1), and 3-4 types (coded 2). The sum of the frequency of exposure for all CM types described the cumulative severity. The scores ranged from 0 (no abuse) to 8 (severe or frequent abuse).

The following mental disorders were measured using international scales which were validated in various internationally settings including South Africa.

Post-traumatic stress disorder (PTSD)/Acute stress reactions (ASR)

PTSD are acute stress reactions to a traumatic event that are sustained beyond 1-month post the event. The baseline assessment among the women who experienced rape were done within 20 days of the incident rape and PTSD due to the rape can therefore not be ascertained and referred to as acute stress reactions.[21] The validated Davidson Trauma self-rating scale was used to measure ASR which included 30 items.[22] Apart from being validated internationally, the scale has been used in other contexts in South Africa. [23,24] Participants were asked how much each item/symptom affected them over the past week, with responses evaluated on a 5-point Likert scale: "not at all" (0), "only once" (1), "2-3 times" (2), "4-6 times" (3) and 'all the time' (4). Scores for all items were summed for an ASR score.

Current depressive symptoms were measured using the 20-item Center for Epidemiologic Studies Depression (CESD) self-report measure,[25] which has been validated in international settings including South Africa.[24,26] The CESD scale was designed to assess how often a person experienced depressive symptoms within the past week. The response was categorized into a 4-point Likert scale ranging from "rarely or none of the time" (0) to "most or all of the times"

For peer review only

(3). The summed score provided the overall CESD score with higher scores indicating more severe depressive symptoms.

Exposures to other prior traumatic events were assessed using an adapted Life Event Checklist.[23,24,27] The checklist includes 10 items of traumatic events namely imprisonment, civil unrest/war, serious injury, being close to death, witnessing a murder of family or friend, unnatural death of family or friend, witnessing the murder of stranger, torture, robbed or carjacked at gun or knife point and kidnapping. The yes (score 1)/no (score 0) scores for all the items were totaled to create a continuous variable of "other traumatic exposures". A higher score was indicative of more lifetime traumatic events or exposures.

Clinical parameters

Anthropometric measures were done using standardized methods. Height was measured to the nearest millimetre with the participant in an upright position and bare-footed. Weight was taken to the nearest 0.1 kg with the participants in light clothing and without shoes. BMI was calculated as weight in kilograms divided by height in metres squared, and BMI $\geq$25kg/m^2 was used to define overweight and obese. Waist circumference (WC) was recorded to the nearest millimetre at the level that is mid-way between the upper border of the hip bone and the lower border of the lowest rib. Elevated WC was defined as WC >80cm.[28]

Blood pressure (BP) was measured using a digital BP monitor (Omron, M6 Comfort, Netherland) after the participant was seated in a resting position for at least five minutes; three measurements were taken three minutes apart, and the average of the 2nd and 3rd readings were used in the analysis. Hypertension was defined as systolic BP $\geq$140mmHg or diastolic BP $\geq$90mmHg or self-reported previous diagnosis of hypertension.[3]

Biochemical parameters

Biochemical measurements were done at an accredited pathology laboratory (Global Laboratory, Durban, South Africa). Serum lipids were analysed by enzymatic colorimetric method; HbA1c was measured using high-performance liquid chromatography (VARIANT II TURBO. EDTA tubes) following the National Glycathaemoglobin Standardisation Programme (NGSP). Dyslipidaemia was based on the South African national lipid guidelines and defined as follows: total cholesterol >5 mmol/L, low-density lipoprotein cholesterol >3 mmol/L,

For peer review only - http://bmjopen.bmj.com/site/about/guidelines.xhtml

triglycerides >1.5 mmol/L and high-density lipoprotein cholesterol <1.2 mmol/L.[29] Dysglycaemia was defined as HbA1c ≥5.7% and or previously diagnosed with diabetes.[30]

Statistical analyses

Data were analyzed using R statistical software version 3.6.0 (2019-04-26). Since this study aimed to determine the associations of CM with adult hypertension in both rape-exposed and non-rape-exposed women, and not to determine and/or compare the prevalence of CM and outcome hypertension between the two groups of women; this analysis used the baseline data from the pooled group of women and not just the women who experienced rape. Exploratory analyses revealed no significant interaction effect of 'rape exposure' on the association of CM with hypertension status, motivating the pooled analyses of both groups (women who experienced rape and women who did not) to maximize the statistical power. This was done using logistic regression models where the interaction term (CM*Rape-exposure) was incorporated in the same model for the effects of CM on outcome hypertension.

The baseline characteristics were summarized as means (standard deviation, SD) or medians (25th to 75th percentiles) for continuous variables, and as count (percentages) for categorical variables in the overall sample and by hypertension status. Comparison of baseline characteristics by hypertension were using chi-square tests, fisher-exact test, t-tests or Kruskal-Wallis tests for non-parametric data where appropriate. The relations of CM with hypertension were explored across 1) each of the four CM categories, 2) multiple combined CM categories and 3) cumulative frequency and severity of CM. The associations were explored using multiple logistic regression analyses with adjustments for potential confounders and mediators. The potential confounders were identified as those variables which were likely correlated with CM and causally linked to the development of hypertension and included age, the recent rape exposure, education level, employment status and residence type.

The potential mediators were based on the existing literature and included BMI, HbA1c, smoking, alcohol drinking, HIV infection, depressive symptoms, ASR and other traumatic event experiences. They were postulated to be involved in the pathways linking CM with hypertension. HIV infection was considered as a potential mediator because previous studies

For peer review only - http://bmjopen.bmj.com/site/about/guidelines.xhtml

in South Africa have shown CM to be associated with HIV-related risk behaviour[16], and living with HIV infection was associated higher risk for hypertension[31]. On rare occasions, HIV infection could have been acquired before CM exposure, but the number would likely be too few to bias the mediation analyses. Other traumatic event experience was positioned as a potential mediator based on the local literature[32]. Although the exact order of occurrence between CM and other traumatic event exposure could not be specified in the current study, CM, by definition, typically occurred only in childhood. Some traumatic events could have happened at any time during the lifespan, and have been conventionally measured through scores that capture the lifetime experience; which were used in the current study. Lifetime experience of these other traumatic events could have been the consequence of CM or not; but CM would very unlikely be the consequence of other traumatic event exposure.

Variables not found to be associated with hypertension in the bivariate analysis ($p > 0.05$) were subsequently excluded from the regression models. The latter included education level, employment status and residence type. Rape exposure variable remained in the models as adjustment variable because it forms part of study design.

The mediation effects of possible mediators adjusted for potential confounders (age and rape-exposure) on the association of CM (any CM, multiple combined CM types and the cumulative severity score) with hypertension were explored using simple mediation analysis.[33] Each mediator was analysed separately in individual mediation models, which comprised the hypertension outcome variable, a predictor (CM exposure) and a mediator. *Laavan* package (http://CRAN.R-project.org/package=lavaan) was used to compute the indirect, direct and total effects estimates, and proportions of mediation effects (indirect effect/total effect), with the significance of the mediation effect tested via bootstrap methods, based on 5000 replications. Estimates are reported as standardized beta coefficients which were derived based on diagonal weighted least square method, but also as odd ratios (OR) and 95% confidence intervals. It is of note, however, that the interpretation of OR from probit models (implemented for mediation analysis in *Laavan*) is not straightforward. We further explored the mediating effects using multiple mediation model with the same adjustment variables. A p-value <0.05 was considered a statistically significant mediation effect. Full or complete mediation is present when the total and indirect effects

For peer review only - http://bmjopen.bmj.com/site/about/guidelines.xhtml

are significant while the direct effect is non-significant. Partial mediation occurs when the total and indirect effects are significant, and the direct effect remains significant.

Furthermore, we have replicated both simple and multiple mediation analyses using 'mediation' package (http://CRAN.R-project.org/package=mediation), and presented the findings as secondary analyses.[34]

Patient and public involvement

In this study, the research questions and outcomes were not informed by patients' priorities, experiences, and preferences. Patient and the public were not involved in the design or conduct or reporting or dissemination plan.

RESULTS

A total of 1799 women were recruited into the study. Two participants had missing data on CM and were excluded. Therefore, the final sample consisted of 1797 participants. Of these, 220 (12.2%) had prevalent hypertension. The characteristics of the participants are presented in Table 1. The overall median age was 24 years; participants with hypertension were older compared to their counterparts without hypertension (26 years vs. 24 years, $p<0.001$). Compared to those without hypertension, women with hypertension were more likely to be HIV-positive and to currently drink alcohol; they had higher scores for depression, ASR, and were more likely to have experienced other traumatic events in their lives (all $p \leq 0.05$). Participants with hypertension had higher mean WC and BMI levels and dysglycaemia, but their lipid profiles were similar to those without hypertension. There was no difference in terms of education, residence, current rape exposure or smoking status between the participants with and without hypertension (Table 1).

For peer review only

Prevalence of CM types by hypertensive status

The distribution of CM types overall and by hypertension status is presented in Table 2 ~~3~~. The prevalence of CM was 58.9% in overall sample, with physical abuse being the most frequent (42.7%). This was followed by emotional abuse and parental neglect at 29% each, while exposure to sexual abuse was 13.4%. The prevalence of all four CM types was significantly higher in participants with, than without, hypertension. The prevalence of CM was 70.9% in

For peer review only - http://bmjopen.bmj.com/site/about/guidelines.xhtml

women with vs. 57.2% in those without hypertension (p<0.001). More than half the women with hypertension (51.8%) had experienced physical abuse compared to 41.5% of women without hypertension (p=0.004). A fifth (20.9%) vs. 12.4% of women with compared to without hypertension were exposed to sexual abuse in childhood. Exposures to 1-2 types and 3-4 types of CM were 46.4% and 24.5%, respectively, in women with hypertension, and lower in women without (42.9% and 14.3%, respectively) (p<0.001). Similar patterns of higher prevalence rates in women with compared to without hypertension were observed for the severity and frequency of abuse experienced (all p≤0.003). There was no evidence of a statistically significant interaction effect of rape exposure on the distribution of CM by hypertension status as shown in Table 3 (all interaction p≥0.061).

Associations of CM exposure with prevalent hypertension

The odds ratios for the relationship of CM with hypertension adjusted for age, BMI, current smoking, current alcohol use, recent rape exposure, HIV infection, other trauma exposure, depression scores, ASR scores and dysglycaemia are presented in Table 3. There was a positive association of exposure to any CM (adjusted odds ratio (AOR): 1.62; 95%CI: 1.19-2.25), sexual abuse (AOR: 1.64; 1.12-2.37), physical abuse (AOR: 1.43; 95%CI: 1.07-1.92) and emotional abuse (AOR: 1.57; 1.16-2.13) with prevalent hypertension. The association of exposure to parental neglect (AOR: 1.37; 1.00-1.86) was borderline. Increasing number of maltreatment types as well as cumulative severity/frequency of maltreatment overall or by maltreatment type were associated with increased odds of hypertension (Table 4).

The results of the mediation analysis showed that the associations of CM (any CM, multiple combined CM types and the cumulative severity score) with hypertension were partially mediated by current alcohol consumption (all p≤0.043 for the mediation effects), depressive symptom score (all p≤0.022 for the mediation effects) and experiencing other traumatic events in life (all p<0.001 for the mediation effects) (Table 5 and Table S1). ASR score partially mediated the association of any CM with hypertension (p=0.024 for the mediation effect) and of multiple CM types (p=0.045 for the mediation effect) but not of the cumulative severity abuse score (p=0.066 for the mediation effect). BMI, HbA1c, HIV status, smoking habit had no significant mediation effects on the association between CM exposure and hypertension (all p≥0.144 for the mediation effects).

For peer review only - http://bmjopen.bmj.com/site/about/guidelines.xhtml

When the multiple mediation analysis model adjusted for age and rape exposure were applied (Tables S2 and S3), the direct effects of any CM (p=0.084 for the direct effect) were completely mediated by current alcohol consumption (p=0.034 for mediation effect), depressive symptom score (p=0.040) and experiencing other traumatic events (p<0.001). The direct effects of multiple CM types and the cumulative severity abuse score on hypertension remained significant, and the associations were partial mediated by current alcohol consumption and experiencing other traumatic events (Figure 1).

Results from secondary analyses of both simple and multiple mediation models showed similar patterns (Tables S3 and S4). In simple mediation models, the associations of any CM and multiple combined CM types with hypertension were partially mediated by current alcohol consumption (both ps≤0.036 for the average causal mediation effects (ACMEs), depressive symptom score (both ps≤0.026 for the ACMEs) and lifetime experiences of other traumatic events (both ps<0.001 for the ACMEs) (Table S3). The associations of the cumulative severity score with hypertension were partially mediated by lifetime experiences of other traumatic events (p<0.001 for the ACME). ASR, BMI, HbA1c, HIV status, and smoking habit had no significant mediation effects on the association between CM exposure and hypertension. In multiple mediation models, the association of any CM with hypertension was partially mediated by depressive symptom score and lifetime experiences of other traumatic events (Table S4). The associations of multiple combined CM types and the cumulative severity score with hypertension were partially mediated by lifetime experiences of other traumatic events. The results of sensitivity analyses suggest that the point estimates of the ACME are rather sensitive to the violation of the sequential ignorability assumption (Figures S1 and S2).

For peer review only

DISCUSSION

In this study among South African adult women attending specific healthcare services, CM was widespread and associated with hypertension. To our knowledge, this is among very few studies conducted in low- and middle-income countries, which describes significant associations between CM and hypertension in women after adjusting for traditional hypertension risk factors, other traumatic exposures and poor mental health. The study showed exposure to sexual, physical, emotional abuse, multiple (3-4) abuse types and

For peer review only - http://bmjopen.bmj.com/site/about/guidelines.xhtml

frequent and severe exposure were significantly associated with hypertension. Further it showed that the associations were potentially mediated by alcohol use, other trauma exposure, depression, and ASR.

These findings accord with the literature from high-income countries; frequent and severe exposure to physical, emotional and sexual abuse and parental neglect in childhood, and exposure to multiple abuse types compared to lesser abuse exposures may have greater negative health consequences,[35] including hypertension.[36,37] The latter includes two longitudinal studies from the United States. Among 394 men and women followed for 23-years, those with multiple CM exposures showed a greater increase in adult BP levels compared to those without childhood traumatic experiences.[36] The Nurses' Health Study II showed that severe childhood physical and/or sexual abuse was associated with an increased risk of hypertension in adulthood independent of race, oral contraceptive use, BMI, smoking, alcohol use, exercise and child somatogram score.[37]

However, these two studies, unlike the current study, did not investigate the effects of mental health issues such as depression and ASR, on the relationship between CM and hypertension. Notably, the current study found that the associations of CM with hypertension remained after adjusting for poor mental health-related factors for all abuse types. Current depressive symptom, ASR and other prior traumatic events mediated the relationships between CM and hypertension in this study. The additive influences of these psychological factors likely demonstrate their role in the development of hypertension. While a detailed exploration of these associations is beyond the scope of this paper, the World Mental Health Survey reported similar findings. The presence of three or more childhood adversities (abuse, neglect, other trauma experiences) was modestly associated with self-reported hypertension, with the association not fully explained by early-onset or current depression-anxiety.[38] In addition, a survey in Brazil found childhood physical abuse and family violence were associated with a greater likelihood of adult hypertension; the association was attenuated by the onset of depression in adulthood.[13] Although CM was associated with hypertension independent of current mental health disorders in the current study, it is important to investigate whether the association is reversible. Such understanding will help to design interventions to prevent the development of hypertension in the CM victims.

For peer review only - http://bmjopen.bmj.com/site/about/guidelines.xhtml

While exposures to any CM, sexual abuse and emotional abuse were significantly associated with hypertension, there was weaker effect of physical abuse or parental neglect with hypertension in the present study. More research, particularly from longitudinal studies, is required for a deeper understanding of these associations and to examine the differential impact of the various types of CM on the development of hypertension. Similar to these findings, a prospective childhood study in the United States reported no significant differences in the associations of physical abuse and neglect with the development of hypertension after 30 years of follow-up.[39] However, unlike the current study, they also found no association between childhood sexual abuse and hypertension. The study controlled for age, gender and race but did not assess the severity of CM.

Another study that examined childhood physical and verbal abuse and neglect using a short-form family environment questionnaire found an indirect association of adverse family environment in childhood with changes of BP over a ten-year period through negative emotions of depression, anxiety or anger.[40] The latter study is not directly comparable to the current study because the questionnaire used only partially addressed verbal and physical abuse and neglect with these assessed as a composite variable.

Additionally, alcohol use partly mediated the relationship between CM and hypertension in the present study. This is in keeping with a systematic review that suggested the association of CM with hypertension may also be influenced by the traditional behavioural risk factors for hypertension.[7] The uptake of risky behaviours were suggested to be coping mechanisms when faced with stressful situations.[5] However, apart from alcohol intake, other traditional risk factors for hypertension such as adiposity, assessed using BMI, did not mediate the associations of CM with hypertension in the RICE study. This may be because the participants in our study were relatively young. Furthermore, our sample was on average overweight-to-obese, restraining the distribution of markers of adiposity in this population. This in turn could affect any reliable investigation of the relationship of adiposity with hypertension in this sample.

Strengths and limitations

The BP of study participants was objectively measured during data collection, unlike other relevant studies that used a self-reported measure of hypertension only.[13,][36],[37] This

For peer review only - http://bmjopen.bmj.com/site/about/guidelines.xhtml

study, therefore, likely captured all participants with hypertension instead of only those who were aware of their hypertension status. Another strength is that this study comprehensively assessed CM by examining four CM types as well as the frequency and severity of the abuse using a well-established locally validated measure.[8] Our study is one of few studies that has assessed the associations of multiple CM types and their frequency and severity with hypertension. This paper, therefore, provides a comprehensive understanding of the relationship between CM and adult hypertension.

The present study has several limitations. The cross-sectional design precludes a reliable quantitative interpretation of the effect of the exposure of interest, the mediation effect and the inferences about causality. However, the median age (26 years, 25th-75th: 23-30) of women with hypertension indicates most hypertension onset occurred in adulthood, whereas CM happened in early life. It is likely that this study may have established the temporal relationships between CM and hypertension. It is also known that retrospective self-report of CM experienced could be subject to under reporting. Although many factors could impact underreporting of childhood experiences, suppressed memory of traumatic experiences have been suggested as a key factor.[41]

Furthermore, BP was measured only on a single occasion, which is not a formal diagnosis of hypertension and tends to over-estimate hypertension. Another limitation is that the volunteer sample of women is not representative of all women in the general population. The data were collected only in women and might not be applicable to men. The non-significant influences of BMI on the associations of CM with prevalent hypertension need to be viewed with caution. It could be that our study population was young (median age 24 years, 25th-75th: 21-29), in whom the traditional risk factors may yet influence the development of hypertension. Considering that the RICE study is a longitudinal study, the follow-up data may demonstrate the influence of traditional risk factors on the associations of CM with hypertension later in the life of these women.

CONCLUSIONS

CM is a serious public health problem and associated with adult hypertension independent of traditional risk factors, other traumatic events, and mental ill-health in young South African women. The associations of CM with hypertension were found to be greater in

For peer review only - http://bmjopen.bmj.com/site/about/guidelines.xhtml

women with multiple maltreatment types and frequent and severe maltreatment, and were partially mediated by alcohol use, current depression, ASR and other traumatic exposures. These findings suggest that prevention of CM is important, not only in itself, but also for the prevention of adult hypertension. Where the prevention of CM is not possible, counselling to manage stress and improve coping skills are important to curb the development of hypertension. This is in line with the goals of psychological therapy for CM survivors which aim to reduce the subsequent health impact. Screening for hypertension in individuals suffering from CM may help the early identification and treatment of the condition. Further, counselling for the other traumatic exposures, and management of depressive symptoms and ASR are also required and may contribute partially to curbing hypertension.

Contributors: KAN led the analysis and drafted the paper. NP, NA, RJ and APK co-conceived and designed the paper. APK, NP, NA, RJ and SM co-conceived the broader study, commented on all drafts, and approved the final submission. APK assisted with the statistical analysis.

Funding: The RICE study was funded by South African Medical Research Council: Flagships Awards Project SAMRC-RFA-IFSP-01-2013. The work reported herein was made possible through funding by the South African Medical Research Council through its Division of Research Capacity Development under the SAMRC Postdoctoral Programme from funding received from the South African National Treasury. The content hereof is the sole responsibility of the authors and do not necessarily represent the official views of the SAMRC or the funders.

Competing interests: The authors declare no conflict of interest.

Patient consent for publication: Not required.

Ethics approval: The South African Medical Research Council Research Ethics Committee provided ethical approval for this study (SAMRC; EC019-10/2013), and all participants provided informed consent.

Data sharing statement: Data are available on request.

REFERENCES

1 World Health Organization. A global brief on hypertension : silent killer, global public health

For peer review only

For peer review only - http://bmjopen.bmj.com/site/about/guidelines.xhtml

crisis: World Health Day 2013. World Heal. Organ. 2013;:40.

2 GBD 2015 Risk Factors Collaborators. Global, regional, and national comparative risk assessment of 79 behavioural, environmental and occupational, and metabolic risks or clusters of risks, 1990-2015: a systematic analysis for the Global Burden of Disease Study 2015. *Lancet (London, England)* 2016;**388**:1659–724. doi:10.1016/S0140-6736(16)31679-8

3 World Health Organization. Hypertension. *World Heal Organ* Published Online First: 2019.https://www.who.int/health-topics/hypertension/#tab=tab_1

4 Cuevas AG, Williams DR, Albert MA. Psychosocial Factors and Hypertension: A Review of the Literature. *Cardiol Clin* 2017;**35**:223–30. doi:10.1016/j.ccl.2016.12.004

5 Wilson DK, Kliewer W, Sica DA. The relationship between exposure to violence and blood pressure mechanisms. *Curr Hypertens Rep* 2004;**6**:321–6. doi:10.1007/s11906-004-0028-2

6 Hillis S, Mercy J, Amobi A, *et al.* Global Prevalence of Past-year Violence Against Children: A Systematic Review and Minimum Estimates. *Pediatrics* 2016;**137**:e20154079. doi:10.1542/peds.2015-4079

7 Hughes K, Bellis MA, Hardcastle KA, *et al.* The effect of multiple adverse childhood experiences on health: a systematic review and meta-analysis. *Lancet Public Heal* 2017;**2**:e356–66. doi:10.1016/S2468-2667(17)30118-4

8 Jewkes RK, Dunkle K, Nduna M, *et al.* Associations between childhood adversity and depression, substance abuse and HIV and HSV2 incident infections in rural South African youth. *Child Abuse Negl* 2010;**34**:833–41. doi:10.1016/j.chiabu.2010.05.002

9 Trickett PK, Noll JG, Putnam FW. The impact of sexual abuse on female development: lessons from a multigenerational, longitudinal research study. *Dev Psychopathol* 2011;**23**:453–76. doi:10.1017/S0954579411000174

10 Norman RE, Byambaa M, De R, *et al.* The long-term health consequences of child physical abuse, emotional abuse, and neglect: a systematic review and meta-analysis. *PLoS Med* 2012;**9**:e1001349. doi:10.1371/journal.pmed.1001349

11 Basu A, McLaughlin KA, Misra S, *et al.* Childhood Maltreatment and Health Impact: The Examples of Cardiovascular Disease and Type 2 Diabetes Mellitus in Adults. *Clin Psychol a Publ Div Clin Psychol Am Psychol Assoc* 2017;**24**:125–39. doi:10.1111/cpsp.12191

12 Anderson EL, Fraser A, Caleyachetty R, *et al.* Associations of adversity in childhood and risk factors for cardiovascular disease in mid-adulthood. *Child Abuse Negl* 2018;**76**:138–48. doi:10.1016/j.chiabu.2017.10.015

13 Parrish C, Surkan PJ, Martins SS, *et al.* Childhood adversity and adult onset of hypertension and heart disease in São Paulo, Brazil. *Prev Chronic Dis* 2013;**10**:E205. doi:10.5888/pcd10.130193

14 National Department of Health (NDoH), Statistics South Africa (Stats SA), South African Medical Research Council (SAMRC) and I. South Africa Demographic and Health Survey 2016. *Pretoria, South Africa, Rockville, Maryland, USA NDoH, Stats SA, SAMRC, ICF* 2019.

15 Meinck F, Cluver LD, Boyes ME, *et al.* Physical, emotional and sexual adolescent abuse victimisation in South Africa: prevalence, incidence, perpetrators and locations. *J Epidemiol Community Health* 2016;**70**:910–6. doi:10.1136/jech-2015-205860

16 Gibbs A, Dunkle K, Washington L, *et al.* Childhood traumas as a risk factor for HIV-risk behaviours amongst young women and men living in urban informal settlements in South

For peer review only - http://bmjopen.bmj.com/site/about/guidelines.xhtml

Africa: A cross-sectional study. *PLoS One* 2018;**13**:e0195369. doi:10.1371/journal.pone.0195369

17 Ward CL, Artz L, Leoschut L, *et al.* Sexual violence against children in South Africa: a nationally representative cross-sectional study of prevalence and correlates. *Lancet Glob Heal* 2018;**6**:e460–8. doi:10.1016/S2214-109X(18)30060-3

18 Abrahams N, Seedat S, Lombard C, *et al.* Study protocol for a longitudinal study evaluating the impact of rape on women's health and their use of health services in South Africa. *BMJ Open* 2017;**7**:e017296. doi:10.1136/bmjopen-2017-017296

19 Abrahams N, Mhlongo S, Chirwa E, *et al.* Rape survivors in South Africa: analysis of the baseline socio-demographic and health characteristics of a rape cohort. *Glob Health Action* 2020;**13**:1834769. doi:10.1080/16549716.2020.1834769

20 Bernstein DP, Stein JA, Newcomb MD, *et al.* Development and validation of a brief screening version of the Childhood Trauma Questionnaire. *Child Abuse Negl* 2003;**27**:169–90. doi:10.1016/s0145-2134(02)00541-0

21 Kleber RJ. Trauma and Public Mental Health: A Focused Review. *Front psychiatry* 2019;**10**:451. doi:10.3389/fpsyt.2019.00451

22 Zlotnick C, Davidson J, Shea MT, *et al.* Validation of the Davidson Trauma Scale in a sample of survivors of childhood sexual abuse. *J Nerv Ment Dis* 1996;**184**:255–7. doi:10.1097/00005053-199604000-00010

23 Ward CL, Flisher AJ, Zissis C, *et al.* Exposure to violence and its relationship to psychopathology in adolescents. *Inj Prev* 2001;**7**:297–301. doi:10.1136/ip.7.4.297

24 Myer L, Smit J, Roux L Le, *et al.* Common mental disorders among HIV-infected individuals in South Africa: prevalence, predictors, and validation of brief psychiatric rating scales. *AIDS Patient Care STDS* 2008;**22**:147–58. doi:10.1089/apc.2007.0102

25 Nduna M, Jewkes RK, Dunkle KL, *et al.* Associations between depressive symptoms, sexual behaviour and relationship characteristics: a prospective cohort study of young women and men in the Eastern Cape, South Africa. *J Int AIDS Soc* 2010;**13**:44. doi:10.1186/1758-2652-13-44

26 Chishinga N, Kinyanda E, Weiss HA, *et al.* Validation of brief screening tools for depressive and alcohol use disorders among TB and HIV patients in primary care in Zambia. *BMC Psychiatry* 2011;**11**:75. doi:10.1186/1471-244X-11-75

27 Gray MJ, Litz BT, Hsu JL, *et al.* Psychometric properties of the life events checklist. *Assessment* 2004;**11**:330–41. doi:10.1177/1073191104269954

28 Alberti KGMM, Eckel RH, Grundy SM, *et al.* Harmonizing the metabolic syndrome: a joint interim statement of the International Diabetes Federation Task Force on Epidemiology and Prevention; National Heart, Lung, and Blood Institute; American Heart Association; World Heart Federation; International . *Circulation* 2009;**120**:1640–5. doi:10.1161/CIRCULATIONAHA.109.192644

29 Nadine Butler. National Guidelines at a glance : hypercholesterolaemia : review. *SA Pharm J* 2009;**76**:22–33.

30 World Health Organization. *Use of Glycated Haemoglobin (HbA1c) in the Diagnosis of Diabetes Mellitus: Abbreviated Report of a WHO Consultation*. Geneva: 2011.

31 Davis K, Perez-Guzman P, Hoyer A, *et al.* Association between HIV infection and hypertension:

For peer review only - http://bmjopen.bmj.com/site/about/guidelines.xhtml

For peer review only

a global systematic review and meta-analysis of cross-sectional studies. *BMC Med* 2021;**19**:105. doi:10.1186/s12916-021-01978-7

32 Gass JD, Stein DJ, Williams DR, *et al.* Intimate partner violence, health behaviours, and chronic physical illness among South African women. *S Afr Med J* 2010;**100**:582–5. doi:10.7196/samj.4274

33 Rosseel Y. lavaan: An R Package for Structural Equation Modeling. *J Stat Softw* 2012;**48**:36.

34 Imai K, Keele L, Yamamoto T. Identification, Inference and Sensitivity Analysis for Causal Mediation Effects. *Stat Sci* 2010;**25**. doi:10.1214/10-STS321

35 Gilbert R, Widom CS, Browne K, *et al.* Burden and consequences of child maltreatment in high-income countries. *Lancet (London, England)* 2009;**373**:68—81. doi:10.1016/s0140-6736(08)61706-7

36 Su S, Wang X, Pollock JS, *et al.* Adverse childhood experiences and blood pressure trajectories from childhood to young adulthood: the Georgia stress and Heart study. *Circulation* 2015;**131**:1674–81. doi:10.1161/CIRCULATIONAHA.114.013104

37 Riley EH, Wright RJ, Jun HJ, *et al.* Hypertension in adult survivors of child abuse: observations from the Nurses' Health Study II. *J Epidemiol Community Health* 2010;**64**:413–8. doi:10.1136/jech.2009.095109

38 Stein DJ, Scott K, Haro Abad JM, *et al.* Early childhood adversity and later hypertension: data from the World Mental Health Survey. *Ann Clin psychiatry Off J Am Acad Clin Psychiatr* 2010;**22**:19–28.

39 Widom CS, Czaja SJ, Bentley T, *et al.* A prospective investigation of physical health outcomes in abused and neglected children: new findings from a 30-year follow-up. *Am J Public Health* 2012;**102**:1135–44. doi:10.2105/AJPH.2011.300636

40 Lehman BJ, Taylor SE, Kiefe CI, *et al.* Relationship of early life stress and psychological functioning to blood pressure in the CARDIA study. *Health Psychol* 2009;**28**:338–46. doi:10.1037/a0013785

41 Widom, C. S., & Morris S. Accuracy of adult recollections of childhood victimization, Part 2: Childhood sexual abuse. *Psychol Assess* 1997;**9**:34–46.

For peer review only

For peer review only - http://bmjopen.bmj.com/site/about/guidelines.xhtml

Table 1: Socio-demographic characteristics and cardiovascular disease risk factors presented by hypertension status

Variables	Overall (N=1797)	Hypertension (n=220)	No hypertension (n=1577)	p-value
Age in years, median (P25-P75)	24 (21-29)	26 (23-30)	24 (21-28)	<0.001
Women who experienced rape	47.4 (45-50)	53.6 (46.8-60.4)	46.4 (39.6-53.2)	0.054
**Years schooling, % (95%CI)**				0.415
1-7 years	2.8 (1.4-4.2)	3.6 (0.4-7.6)	2.7 (1.2-4.1)	
8-12 years	89 (87.6-90.4)	90 (86.8-93.9)	88.8 (87.4-90.3)	
>12 years	8.2 (6.9-9.6)	6.4 (3.2-10.3)	8.5 (7-10)	
**Employment status, % (95%CI)**				
Employed, **% (95%CI)**	20.5 (18.6-22.4)	24.1 (18.6-30.3)	20 (18-22)	0.184
**Residence, % (95%CI)**	**N=1774**	**n=218**	**n=1556**	0.790
Formal urban	72 (70-74)	74 (68.3-79.6)	71.7 (69.5-74)	
Informal urban	16.6 (14.5-18.7)	15 (9.6-20.9)	16.8 (14.6-19)	
Rural/semi-rural	11.4 (9.4-13.6)	11 (5.5-16.7)	11.5 (9.3-13.8)	
**HIV-positive, % (95%CI)**	43.3 (40.9-45.6)	52.7 (45.9-59.5)	42 (39.5-44.4)	0.003
**Lifetime trauma event exposure:**				
Other traumatic exposures score, median (P25-P75)	2 (1-3)	2 (1-4)	2 (1-3)	<0.001
**Health behaviours, % (95%CI)**				
Current smoker	12.5 (11-14)	13.6 (9.4-18.9)	12.3 (10.7-14)	0.651
Current alcohol consumption	53.1 (50.7-55.4)	61.8 (55-68.2)	51.9 (49.4-54.4)	0.006
**Mental health disorders**				
Depression score	21 (11-36)	20 (11-36)	28 (14-38)	<0.001
ASR score, median (P25-P75)	16 (2-37)	22 (5-41)	15 (1-37)	0.001
**Cardiometabolic health**				
Mean (SD) or Median (P25-P75)				
Systolic blood pressure, mmHg	105 (8.5)	108 (11.2)	104 (7.9)	<0.001
Diastolic blood pressure, mmHg	70 (7.6)	73 (10.3)	70 (7.1)	<0.001
Waist circumference, cm	84 (13.9)	88 (15)	84 (13.7)	<0.001
Body mass index	27 (6.6)	29 (8.3)	27 (6.3)	<0.001
	**N=1614**	**n=169**	**n=1445**	
Total cholesterol, mmol/L	3.8 (0.82)	4.0 (0.84)	3.8 (0.81)	0.052
Low-density lipoprotein cholesterol (LDL-C), mmol/L	2.2 (0.73)	2.3 (0.74)	2.2 (0.72)	0.385
High-density lipoprotein cholesterol (HDL-C), mmol/L	1.2 (0.33)	1.25 (0.37)	1.2 (0.33)	0.128
Triglycerides, mmol/L	0.72 (0.52-0.98)	0.74 (0.55-1.01)	0.72 (0.52-0.98)	0.402
Glycated haemoglobin (HbA1c), %	5.3 (0.6)	5.4 (0.7)	5.3 (0.5)	0.169
Prevalence, % (95%CI)				
Waist circumference ≥80cm	57.6 (55.3-59.9)	64.1 (57.4-70.4)	56.7 (54.3-59.2)	0.046
Body mass index ≥25kg/m^2	54.4 (52.1-56.7)	60.4 (53.7-66.9)	53.6 (51.1-56.1)	0.065
Total cholesterol >5mmol/L	7.5 (6.3-8.9)	8.9 (5.1-14.2)	7.3 (6-8.8)	0.572
LDL-C >3mmol/L	13.5 (11.9-15.3)	16 (10.8-22.4)	13.2 (11.5-15.1)	0.382
HDL-C <1.2mmol/L	53.6 (51.1-56)	49.7 (41.9-57.5)	54 (51.4-56.6)	0.322
Triglycerides >1.5mmol/L	7.2 (6-8.6)	7.1 (3.7-12.1)	7.3 (6-8.7)	0.999
HbA1c ≥5.7%	21.1 (19.2-23.1)	30 (24-36.5)	19.9 (17.9-21.9)	0.001
History of known diabetes	1.4 (0.9-2)	2.7 (1-5.8)	1.2 (0.7-1.9)	0.112
History of known hypertension	11.6 (10.1-13.1)	94.5 (90.7-97.2)	NA	NA

Hypertension defined as follows: systolic blood pressure ≥140mmHg and/or diastolic blood pressure ≥90mmHg or previously diagnosed with hypertension; current smoker: currently smoke any tobacco products such as cigarettes,

For peer review only - http://bmjopen.bmj.com/site/about/guidelines.xhtml

cigars or pipes; current alcohol consumption, consumed at least one drink in an occasion within a month, respectively (one drink is equivalent to one can/bottle of beer, cider, cooler/glass of wine/tot of spirit); severe depression: total scores ≥16 for depressive symptoms during the past week using Centre for Epidemiologic Studies Depression Scale (CES-D score); ASR, acute stress reactions, was measures using Davidson Trauma Scale. P-values for chi-square tests, fisher-exact test, t-tests or Kruskal-Wallis tests for non-parametric data where appropriate.

For peer review only

For peer review only - http://bmjopen.bmj.com/site/about/guidelines.xhtml

Table 2: Prevalence of childhood maltreatment types overall and by hypertension status

Number, (%)	Overall (N=1797)			Hypertension (n=220)			No hypertension (n=1577)			P-value
	n	%	95%CI	n	%	95%CI	n	%	95%CI	
**Ever exposed to childhood maltreatment**										
Sexual abuse	241	13.4	11.9-15.1	46	20.9	15.7-26.9	195	12.4	10.8-14.1	<0.001
Physical abuse	768	42.7	40.4-45.1	114	51.8	41.4-54.9	654	41.5	39-43.9	0.004
Emotional abuse	523	29.1	27-31.3	88	40.0	33.4-46.8	435	27.6	25.4-29.9	<0.001
Parental neglect	482	26.8	24.8-28.9	77	35.0	28.7-41.7	405	25.7	23.5-27.9	0.004
Any childhood maltreatment	1058	58.9	56.5-61.2	156	70.9	64.4-76.8	902	57.2	54.7-59.6	<0.001
**Exposure to multiple types of childhood maltreatment**										<0.001
No abuse	739	41.1	38.6-43.6	64	29.1	22.3-36.4	675	42.8	40.1-45.5	
1-2 types	778	43.3	40.8-45.8	102	46.4	39.5-53.7	676	42.9	40.2-45.5	
3-4 types	280	15.6	13.1-18.1	54	24.5	17.7-31.8	226	14.3	11.7-17	
**Combined frequency and severity of childhood maltreatment**										
**Sexual abuse**										<0.001
Never	1556	86.6	85.1-88.1	174	79.1	74.1-84.2	1382	87.6	86.1-89.2	
Some	156	8.7	7.2-10.2	25	11.4	6.4-16.5	131	8.3	6.8-9.9	
Often	85	4.7	3.3-6.3	21	9.5	4.5-14.7	64	4.1	2.5-5.6	
**Physical abuse**										0.002
Never	1029	57.3	54.9-59.7	106	48.2	41.4-55.4	923	58.5	56.1-61.1	
Some	378	21.0	18.7-23.5	48	21.8	15-29	330	20.9	18.5-23.5	
Often	390	21.7	19.4-24.1	66	30.0	23.2-37.2	324	20.5	18.1-23.1	
**Emotional abuse**										<0.001
Never	1274	70.9	68.8-73	132	60.0	53.6-66.8	1142	72.4	70.3-74.6	
Some	290	16.1	14.1-18.3	44	20.0	13.6-26.7	246	15.6	13.4-17.8	
Often	233	13.0	10.9-15.1	44	20.0	13.6-26.7	189	12.0	9.8-14.2	
**Parental neglect**										0.003
Never	1315	73.2	71.2-75.2	143	65.0	59.1-71.6	1172	74.3	72.2-76.5	
Some	328	18.3	16.2-20.3	47	21.4	15.4-28	281	17.8	15.7-20	
Often	154	8.6	6.6-10.6	30	13.6	7.7-20.3	124	7.9	5.8-10	

Frequency and severity of childhood maltreatment defined as: Some: affirmative response of "sometimes" to 1 item only in a specific maltreatment category; Often: affirmative response of "sometime" to >1 item, or response of "often" or "very often" to at least 1 item in a specific maltreatment category. P-values for chi-square tests or fisher-exact tests where appropriate.

For peer review only - http://bmjopen.bmj.com/site/about/guidelines.xhtml

Table 3: Logistic regression analyses for the associations of childhood maltreatment with hypertension

Childhood maltreatment (CM) variable	Sample size	Hypertension n (%)	Unadjusted Odds Ratio	P-value	P-interaction (CM*RE)	Adjusted Odds Ratio	P-value
**Ever exposed to CM**							
Any CM					0.083		
No	739	64 (8.7)	1.00			1.00	
Yes	1058	156 (14.7)	1.82 (1.35-2.50)	<0.001		1.62 (1.19-2.25)	0.002
Sexual abuse					0.081		
No	1556	174 (11.2)	1.00			1.00	
Yes	241	46 (19.1)	1.87 (1.30-2.66)	<0.001		1.64 (1.12-2.37)	0.009
Physical abuse					0.468		
No	1029	106 (10.3)	1.00			1.00	
Yes	768	114 (14.8)	1.52 (1.14-2.02)	0.004		1.43 (1.07-1.92)	0.016
Emotional abuse					0.201		
No	1274	132 (10.4)	1.00			1.00	
Yes	523	88 (16.8)	1.75 (1.30-2.34)	<0.001		1.57 (1.16-2.13)	0.003
Parental neglect					0.557		
No	1315	143 (10.9)	1.00			1.00	
Yes	482	77 (16.0)	1.56 (1.15-2.09)	0.004		1.37 (1.00-1.86)	0.049
**Exposure to multiple types of CM**					0.101		
No abuse	739	64 (8.7)	1.00			1.00	
1-2 abuse types	778	102 (13.1)	1.59 (1.15-2.22)	0.005		1.41 (1.01-1.99)	0.046
3-4 abuse types	280	54 (19.3)	2.52 (1.70-3.72)	<0.001		1.81 (1.18-2.76)	0.006
**Frequency and severity of CM**							
Cumulative maltreatment (range 0-8)	1797	220 (12.2)	1.19 (1.12-1.28)	<0.001	0.061	1.13 (1.05-1.21)	0.002
Sexual abuse					0.081		
Never	1556	174 (11.2)	1.00			1.00	
Some	156	25 (16.0)	1.52 (0.94-2.35)	0.073		1.39 (0.86-2.19)	0.164
Often	85	21(24.7)	2.61 (1.52-4.30)	<0.001		2.12 (1.21-3.60)	0.007
Physical abuse					0.378		
Never	1029	106 (10.3)	1.00			1.00	
Some	378	48 (12.7)	1.27 (0.87-1.81)	0.202		1.24 (0.85-1.79)	0.246
Often	390	66 (16.9)	1.77 (1.27-2.47)	<0.001		1.62 (1.14-2.27)	0.006
Emotional abuse					0.116		
Never	1274	132 (10.4)	1.00			1.00	
Some	290	44 (15.2)	1.55 (1.06-2.22)	0.020		1.44 (0.97-2.08)	0.061
Often	233	44 (18.9)	2.01 (1.37-2.91)	<0.001		1.75 (1.17-2.57)	0.005
Parental neglect					0.139		
Never	1315	143 (10.9)	1.00			1.00	
Some	328	47 (14.3)	1.37 (0.95-1.94)	0.081		1.21 (0.84-1.73)	0.302
Often	154	30 (19.5)	1.98 (1.26-3.03)	0.002		1.74 (1.09-2.71)	0.016

CM, childhood maltreatment; RE, rape exposure; logistic regression models adjusted for age, BMI, recent rape exposure, current smoking; current alcohol use, HIV infection, dysglycemia (HbA1c≥5.7% and or previously diagnosed with diabetes), other traumatic exposures scores, depression scores and acute stress reactions (ASR) scores. Current smoking: currently

For peer review only - http://bmjopen.bmj.com/site/about/guidelines.xhtml

smoke any tobacco products such as cigarettes, cigars or pipes; current alcohol consumption: consumed at least one drink in an occasion within a month, respectively (one drink is equivalent to one can/bottle of beer, cider, cooler/glass of wine/tot of spirit); depression scores, depressive symptoms during the past week using Centre for Epidemiologic Studies Depression Scale (CES-D score); ASR (scores), acute stress reactions measured using Davidson Trauma Scale, a validated self-rating scale. Cumulative maltreatment: ranged from 0 (no maltreated in any type) to 8 (often/severely maltreated in all 4 types).

For peer review only

For peer review only - http://bmjopen.bmj.com/site/about/guidelines.xhtml

Table 4: Results of simple mediation analysis for the effects of childhood maltreatment on adult prevalent hypertension adjusted for age and rape exposure in a sample of South African women (N=1797)

Confounder/ mediator	Total effect			Direct effect			Indirect effect			Proportions of mediation effects
	Estimates	95% CI	P-value	Estimates	95% CI	P-value	Estimates	95% CI	P-value	% (95%CI)
**Any CM**	0.312	0.151-0484	<0.001							
BMI				0.301	0.140-0.463	<0.001	0.011	-0.004-0.026	0.164	3.5 (-1.6-11)
HbA1c				0.314	0.151-0.473	<0.001	-0.002	-0.015-0.011	0.750	-0.6 (-5.5-1.6)
HIV-positive				0.310	0.148-0.471	<0.001	0.002	-0.014-0.018	0.823	0.9 (2.9-6.3)
Current smoking				0.308	0.144-0.468	<0.001	0.003	-0.029-0.034	0.872	0.5 (-3.8-6.8)
Current alcohol				0.282	0.119-0.444	<0.001	0.029	0.001-0.058	0.049	5.6 (1.2-16.7)
Depressive symptom score				0.282	0.122-0.443	<0.001	0.029	0.004-0.053	0.022	12.5 (3.6-29.5)
ASR score				0.287	0.124-0.449	<0.001	0.024	0.008-0.055	0.045	10.5 (1.5-29)
Other traumatic exposures score				0.232	0.067-0.397	0.006	0.081	0.045-0.118	<0.001	27.2 (16.9-68.6)
**Multiple CM types**	0.243	0.138-0.349	<0.001							
BMI				0.236	0.130-0.342	<0.001	0.008	-0.003-0.018	0.157	2.1 (1.5-8.4)
HbA1c				0.244	0.138-0.349	<0.001	-0.001	-0.009-0.007	0.777	0.1 (-3.3-2.2)
HIV-positive				0.238	0.132-0.344	<0.001	0.005	-0.006-0.017	0.374	1.8 (0.9-6.8)
Current smoking				0.240	0.132-0.348	<0.001	0.001	-0.026-0.027	0.959	0.1 (-4.9-5.9)

For peer review only - http://bmjopen.bmj.com/site/about/guidelines.xhtml

Current alcohol				0.223	0.116-0.330	<0.001	0.020	0.001-0.040	0.046	5.6 (0.8-12.4)
Depressive symptom score				0.220	0.113-0.326	<0.001	0.022	0.001-0.043	0.037	11 (3-26)
ASR score				0.226	0.119 - 0.333	<0.001	0.016	0.001-0.039	0.051	9.2 (1.0-25.3)
Other traumatic exposures score				0.178	0.069-0.287	0.001	0.066	0.057-0.095	<0.001	30.9 (15-60)
**Cumulative CM score**	0.094	0.055-0.132	<0.001							
BMI				0.092	0.053-0.130	<0.001	0.002	-0.002-0.005	0.363	0.3 (-3.3-6.5)
HbA1c				0.094	0.056-0.133	<0.001	-0.001	-0.004 – 0.001	0.347	-0.4 (-4.2-1.2)
HIV-positive				0.092	0.054-0.131	<0.001	0.001	-0.004-0.005	0.704	1.1 (-1.4-5.5)
Current smoking				0.092	0.053-0.132	<0.001	0.000	-0.010 – 0.011	0.994	-0.4 (-5.1-6)
Current alcohol				0.086	0.048-0.126	<0.001	0.007	0.001-0.014	0.049	4.5 (0.6-12)
Depressive symptom score				0.084	0.045-0.123	<0.001	0.008	0.001-0.017	0.047	11.1 (2.1-24.8)
ASR score				0.087	0.047-0.126	<0.001	0.006	0.000-0.016	0.193	8.6 (0.8-22.4)
Other traumatic exposures score				0.067	0.027-0.107	0.001	0.027	0.015-0.038	<0.001	31.5 (16.5-65.4)

Data are standardized beta co-efficient. CM, childhood maltreatment; current smoking: currently smoke any tobacco products such as cigarettes, cigars or pipes; current alcohol consumption: consumed at least one drink in an occasion within a month, respectively (one drink is equivalent to one can/bottle of beer, cider, cooler/glass of wine/tot of spirit); depressive symptom (scores): depressive symptoms during the past week using Centre for Epidemiologic Studies Depression Scale (CES-D score); ASR (scores): acute stress reactions measured using Davidson Trauma Scale, a validated self-rating scale. Cumulative abuse (score): ranged from 0 (no abused in any type) to 8 (often/severely abused in all 4 types).

For peer review only - http://bmjopen.bmj.com/site/about/guidelines.xhtml

Table 5: Results of multiple mediation analysis for the effects of childhood maltreatment on adult prevalent hypertension adjusted for age and rape exposure in a sample of South African women (N=1797)

**Independent variables**	**Total effect**			**Direct effect**			**Indirect effect**		
	Estimates	95% CI	P-value	Estimates	95% CI	P-value	Estimates	95% CI	P-value
**Any CM**	0.335	0.166-0.505	<0.001	0.163	-0.022-0.347	0.084			
BMI							0.010	-0.004-0.023	0.154
HbA1c							-0.002	-0.015-0.011	0.768
HIV-positive							0.002	-0.008-0.011	0.694
Current smoking							0.003	-0.030-0.035	0.867
Current alcohol							0.038	0.003-0.073	0.034
Depressive symptom score							0.026	0.001-0.051	0.040
ASR score							0.020	-0.013-0.053	0.232
Other traumatic exposures score							0.076	0.040-0.113	<0.001
**Multiple CM types**									
BMI	0.256	0.146-0.367	<0.001	0.127	0.003-0.251	0.045	0.007	-0.003-0.016	0.160
HbA1c							-0.001	-0.009-0.007	0.872
HIV-positive							0.003	-0.004-0.011	0.414
Current smoking							0.001	-0.026-0.028	0.947
Current alcohol							0.027	0.002-0.052	0.040
Depressive symptom score							0.020	-0.001-0.041	0.067
ASR score							0.012	-0.013-0.038	0.337
Other traumatic exposures score							0.061	0.032-0.090	<0.001
**Cumulative CM score**									
BMI	0.098	0.059-0.138	<0.001	0.052	0.007-0.097	0.024	0.002	-0.002-0.005	0.337
HbA1c							-0.001	-0.004 – 0.002	0.364
HIV-positive							0.001	-0.002-0.003	0.655
Current smoking							0.000	-0.010 – 0.010	0.976
Current alcohol							0.009	0.001-0.018	0.038
Depressive symptom score							0.007	-0.001 – 0.016	0.090
ASR score							0.004	-0.006-0.015	0.425

For peer review only - http://bmjopen.bmj.com/site/about/guidelines.xhtml

Other traumatic exposures score							0.024	0.012-0.037	<0.001

Data are standardized co-efficient. CM, childhood maltreatment; current smoking: currently smoke any tobacco products such as cigarettes, cigars or pipes; current alcohol consumption: consumed at least one drink in an occasion within a month, respectively (one drink is equivalent to one can/bottle of beer, cider, cooler/glass of wine/tot of spirit); depressive symptom (scores): depressive symptoms during the past week using Centre for Epidemiologic Studies Depression Scale (CES-D score); ASR (scores): acute stress reactions measured using Davidson Trauma Scale, a validated self-rating scale. Cumulative abuse (score): ranged from 0 (no abused in any type) to 8 (often/severely abused in all 4 types).

Figure 1: Multiple mediation model of the association of cumulative childhood maltreatment score with adult prevalent hypertension, adjusted for age and rape exposure. All path estimates are standardized regression coefficients. * $p<0.05$, ** $p<0.001$. ASR: acute stress reactions. There are significant indirect paths through current alcohol use: 0.009 (0.001-0.018, p=0.038), through experiencing other trauma: 0.024 (0.012-0.037, p<0.001).

For peer review only

For peer review only - http://bmjopen.bmj.com/site/about/guidelines.xhtml

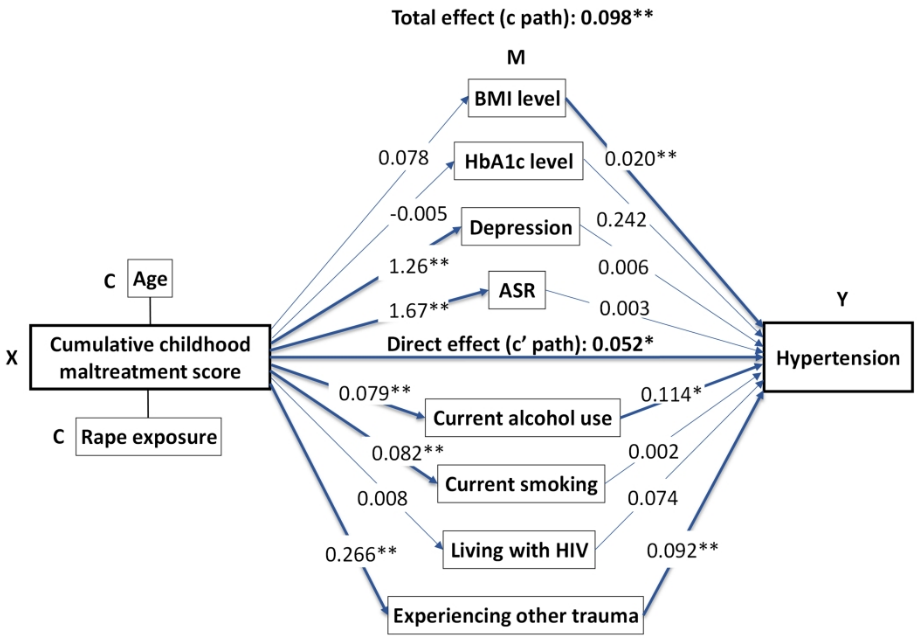

Figure 1: Multiple mediation model of the association of cumulative childhood maltreatment score with adult prevalent hypertension, adjusted for age and rape exposure. All path estimates are standardized regression coefficients. * p<0.05, ** p<0.001. ASR: acute stress reactions. There are significant indirect paths through current alcohol use: 0.009 (0.001-0.018, p=0.038), through experiencing other trauma: 0.024 (0.012-0.037, p<0.001).

Table S1: Results of mediation analysis for the effects of childhood maltreatment on adult prevalent hypertension in a sample of South African women (N=1797)

Independent variables	Total effect			Direct effect			Indirect effect		
	Estimates	95% CI	P-value	Estimates	95% CI	P-value	Estimates	95% CI	P-value
**Any CM**	1.78	1.34-2.43	<0.001						
BMI				1.76	1.33-2.34	<0.001	1.01	0.99-1.01	0.836
HbA1c				1.78	1.35-2.39	<0.001	0.99	0.97-1.02	0.899
HIV-positive				1.77	1.32-2.38	<0.001	1.01	0.97-1.04	0.655
Current smoking				1.76	1.32-2.40	<0.001	1.01	0.94-1.08	0.826
Current alcohol				1.66	1.24-2.78	<0.001	1.07	1.01-1.15	0.036
Depressive symptom score				1.66	1.25-2.25	<0.001	1.07	1.02-1.13	0.007
ASR score				1.67	1.27-2.25	<0.001	1.06	1.01-1.12	0.024
Other traumatic exposures score				1.53	1.14-2.08	0.006	1.18	1.10-1.26	<0.001
**Multiple CM types**	1.58	1.30-1.92	<0.001						
BMI				1.56	1.29-1.89	<0.001	1.01	0.99-1.03	0.363
HbA1c				1.57	1.29-1.93	<0.001	1.00	0.97-1.02	0.948
HIV-positive				1.55	1.28-1.88	<0.001	1.01	0.99-1.10	0.283
Current smoking				1.56	1.29-1.92	<0.001	1.00	0.95-1.06	0.916
Current alcohol				1.50	1.23-1.82	<0.001	1.05	1.01-1.11	0.043
Depressive symptom score				1.55	1.23-1.82	<0.001	1.05	1.01-1.10	0.011
ASR score				1.50	1.24-1.83	<0.001	1.04	1.01-1.09	0.045
Other traumatic exposures score				1.39	1.13-1.70	0.001	1.14	1.08-1.21	<0.001
**Cumulative CM score**	1.19	1.11-1.28	<0.001						
BMI				1.19	1.11-1.28	<0.001	1.00	0.99-1.01	0.829
HbA1c				1.19	1.11-1.28	<0.001	1.00	0.99-1.01	0.500
HIV-positive				1.19	1.11-1.27	<0.001	1.00	0.99-1.01	0.422
Current smoking				1.19	1.11-1.28	<0.001	1.00	0.98-1.02	0.955
Current alcohol				1.17	1.09-1.26	<0.001	1.02	1.01-1.03	0.038
Depressive symptom score				1.17	1.09-1.25	<0.001	1.02	1.01-1.04	0.022
ASR score				1.17	1.09-1.26	<0.001	1.02	1.00-1.04	0.066
Other traumatic exposures score				1.13	1.05-1.21	0.001	1.06	1.03-1.08	<0.001

All models adjusted for age and recent rape exposure; data are odds ratios from probit models. CM, childhood maltreatment; current smoking: currently smoke any tobacco products such as cigarettes, cigars or pipes; current alcohol consumption: consumed at least one drink in an occasion within a month, respectively (one drink is equivalent to one can/bottle of beer, cider, cooler/glass of wine/tot of spirit); depressive symptom (scores): depressive symptoms during the past week using Centre for Epidemiologic Studies

For peer review only

Depression Scale (CES-D score); ASR: acute stress reactions measured using Davidson Trauma Scale, a validated self-rating scale. Cumulative abuse (score): ranged from 0 (no abused in any type) to 8 (often/severely abused in all 4 types).

Table S2: Results of multiple mediation analysis for the effects of childhood maltreatment on adult prevalent hypertension in a sample of South African women (N=1797)

Independent variables	Total effect			Direct effect			Indirect effect		
	Estimates	95% CI	P-value	Estimates	95% CI	P-value	Estimates	95% CI	P-value
**Any CM**	1.84	1.35-2.51	<0.001	1.35	0.96-1.88	0.084			
BMI							1.02	0.99-1.04	0.154
HbA1c							0.99	0.97-1.02	0.768
HIV-positive							1.00	0.98-1.02	0.694
Current smoking							1.01	0.95-1.07	0.867
Current alcohol							1.07	1.01-1.14	0.034
Depressive symptom score							1.05	1.01-1.10	0.040
ASR score							1.04	0.98-1.10	0.232
Other traumatic exposures score							1.14	1.07-1.23	<0.001
**Multiple CM types**	1.59	1.30-1.95	<0.001	1.26	1.01-1.58	0.045			
BMI							1.01	0.99-1.03	0.160
HbA1c							1.00	0.98-1.01	0.872
HIV-positive							1.01	0.99-1.02	0.414
Current smoking							1.00	0.95-1.05	0.947
Current alcohol							1.05	1.01-1.10	0.040
Depressive symptom score							1.04	1.00-1.08	0.067
ASR score							1.02	0.98-1.07	0.337
Other traumatic exposures score							1.12	1.06-1.18	<0.001
**Cumulative CM score**	1.19	1.11-1.29	<0.001	1.10	1.01-1.19	0.024			
BMI							1.00	0.99-1.01	0.337
HbA1c							1.00	0.99-1.01	0.364
HIV-positive							1.00	0.99-1.01	0.655
Current smoking							1.00	0.98-1.02	0.976
Current alcohol							1.02	1.01-1.03	0.038
Depressive symptom score							1.01	1.00-1.03	0.090
ASR score							1.01	0.99-1.03	0.425

For peer review only

Other traumatic exposures score							1.04	1.02-1.07	<0.001

All models adjusted for age and recent rape exposure; data are odds ratios from probit model. CM, childhood maltreatment; current smoking: currently smoke any tobacco products such as cigarettes, cigars or pipes; current alcohol consumption: consumed at least one drink in an occasion within a month, respectively (one drink is equivalent to one can/bottle of beer, cider, cooler/glass of wine/tot of spirit); depressive symptom (scores): depressive symptoms during the past week using Centre for Epidemiologic Studies Depression Scale (CES-D score); ASR: acute stress reactions measured using Davidson Trauma Scale, a validated self-rating scale. Cumulative abuse (score): ranged from 0 (no abused in any type) to 8 (often/severely abused in all 4 types).

For peer review only

Table S3: Results of mediation analysis for the effects of childhood maltreatment on adult prevalent hypertension in a sample of South African women (N=1797).

Confounder/ mediator	Average total effect			Average direct effect			Average indirect effect			Proportion of mediation effect
	Coefficient	95% CI	P-value	Coefficient	95% CI	P-value	Coefficient	95% CI	P-value	% (95%CI)
**Any CM**	0.060	0.030 – 0.090	<0.001							
BMI				0.057	0.028-0.09	<0.001	0.002	-0.000 – 0.01	0.120	4 (-0.3-14)
HbA1c				0.060	0.03-0.09	<0.001	-0.001	-0.003-0.00	0.810	-0.3 (-6.1-10)
HIV-positive				0.059	0.029-0.09	<0.001	-0.001	-0.003-0.00	0.860	-2 (-4.1-6)
Current smoking				0.059	0.03-0.09	<0.001	0.000	-0.003-0.00	0.930	0.3 (-4.9-7)
Current alcohol				0.060	0.026-0.008	<0.001	0.004	0.001-0.01	0.027	6.8 (1.5-21)
Depressive symptom score				0.054	0.024-0.008	<0.001	0.006	0.001-0.01	0.018	10 (2.2-26)
ASR score				0.055	0.026-0.09	<0.001	0.005	0.001-0.01	0.061	8.6 (0.8-28)
Other traumatic exposures score				0.046	0.013-0.08	0.009	0.018	0.011-0.03	<0.001	26.2 (15-70)
**Multiple CM types**	0.046	0.025-0.06	<0.001							
BMI				0.043	0.024-0.06	<0.001	0.002	-0.000 – 0.00	0.130	3.1 (-0.4-10)
HbA1c				0.045	0.025-0.06	<0.001	-0.000	-0.002-0.001	0.850	-0.2 (-4.2-2)
HIV-positive				0.045	0.025-0.06	<0.001	-0.000	-0.001-0.00	0.490	-0.07 (-2.7-9)
Current smoking				0.045	0.025-0.06	<0.001	-0.000	-0.002 – 0.00	0.990	-0.05 (-5.4-6)
Current alcohol				0.043	0.023-0.06	<0.001	0.003	0.000 – 0.01	0.036	6.9 (0.7-17)
Depressive symptom score				0.041	0.021-0.06	<0.001	0.005	0.001-0.01	0.026	11 (1.5-27)
ASR score				0.042	0.023-0.06	<0.001	0.003	-0.001-0.01	0.099	6.2 (-1.2-21)

Other traumatic exposures score				0.033	0.012-0.05	0.002	0.013	0.007-0.02	<0.001	30.5 (18.6-67)
**Cumulative CM score**	0.017	0.011-0.02	<0.001							
BMI				0.016	0.01-0.02	<0.001	0.000	-0.001 – 0.001	0.420	1.9 (-2.4-8)
HbA1c				0.016	0.01-0.02	<0.001	-0.000	-0.001 – 0.00	0.600	-0.7 (-5.1-1)
HIV-positive				0.016	0.01-0.02	<0.001	-0.001	-0.001-0.00	0.890	-3.1 (-10-10)
Current smoking				0.016	(0.01-0.02)	<0.001	-0.000	-0.001 – 0.001	0.910	-0.24 (-6.4-5)
Current alcohol				0.015	0.009 – 0.03	<0.001	0.001	-0.001-0.001	0.280	3.6 (-3.4-13)
Depressive symptom score				0.015	0.01-0.02	<0.001	0.002	0.000 – 0.004	0.056	10 (1-26)
ASR score				0.015	0.010-0.02	<0.001	0.001	-0.001-0.00	0.270	6.1 (-4.3-22)
Other traumatic exposures score				0.012	0.004-0.02	0.003	0.005	0.003-0.010	<0.001	30 (18-66)

All models adjusted for age and recent rape exposure. Data are regression coefficients**;** CM, childhood maltreatment; current smoking: currently smoke any tobacco products such as cigarettes, cigars or pipes; current alcohol consumption: consumed at least one drink in an occasion within a month, respectively (one drink is equivalent to one can/bottle of beer, cider, cooler/glass of wine/tot of spirit); depressive symptom (scores): depressive symptoms during the past week using Centre for Epidemiologic Studies Depression Scale (CES-D score); ASR: acute stress reactions measured using Davidson Trauma Scale, a validated self-rating scale. Cumulative CM (score): ranged from 0 (no maltreated in any type) to 8 (often/severely maltreated in all 4 types).

Table S4: Results of multiple mediation analysis for the effects of childhood maltreatment on adult prevalent hypertension in a sample of South African women (N=1797)

**Independent variables**	**Average total effect**		**Average direct effect**		**Average causal mediation effect**	
	Estimates	95% CI	Estimates	95% CI	Estimates	95% CI
**Any CM**	0.060*	0.030-0.090	0.057*	0.028-0.09		
BMI					0.003	-0.001-0.01
HbA1c					0.000	-0.001-0.00
HIV-positive					0.001	-0.001-0.00
Current smoking					-0.001	-0.005-0.00
Current alcohol					0.004	-0.003-0.01
Depressive symptom score					0.012*	0.002-0.02
ASR score					-0.011	-0.023-0.00
Other traumatic exposures score					0.016*	0.006-0.03
**Multiple CM types**	0.050*	0.028-0.070	0.038*	0.009-0.06		

For peer review only

BMI					0.001	-0.008-0.01
HbA1c					-0.001	-0009-0.01
HIV-positive					-0.000	-0.009-0.01
Current smoking					-0.002	-0.011-0.01
Current alcohol					0.001	-0.007-0.01
Depressive symptom score					0.005	-0.006-0.02
ASR score					-0.006	-0.017-0.00
Other traumatic exposures score					0.014*	0.002-0.03
**Cumulative CM score**	0.021*	0.012-0.03	0.024*	0.013-0.030		
BMI					-0.001	-0.001-0.01
HbA1c					-0.002	-0.008-0.00
HIV-positive					-0.002	-0008-0.01
Current smoking					-0.002	-0.008-0.01
Current alcohol					-0.001	-0.008-0.01
Depressive symptom score					-0.001	-0.008-0.01
ASR score					-0.003	-0.010-0.00
Other traumatic exposures score					0.006*	0.000-0.01

All models adjusted for age and recent rape exposure, data are regression coefficients; CM, childhood maltreatment; current smoking: currently smoke any tobacco products such as cigarettes, cigars or pipes; current alcohol consumption: consumed at least one drink in an occasion within a month, respectively (one drink is equivalent to one can/bottle of beer, cider, cooler/glass of wine/tot of spirit); depressive symptom (scores): depressive symptoms during the past week using Centre for Epidemiologic Studies Depression Scale (CES-D score); ASR: acute stress reactions measured using Davidson Trauma Scale, a validated self-rating scale. Cumulative CM (score): ranged from 0 (no maltreated in any type) to 8 (often/severely maltreated in all 4 types). *, statistical significance.

For peer review only

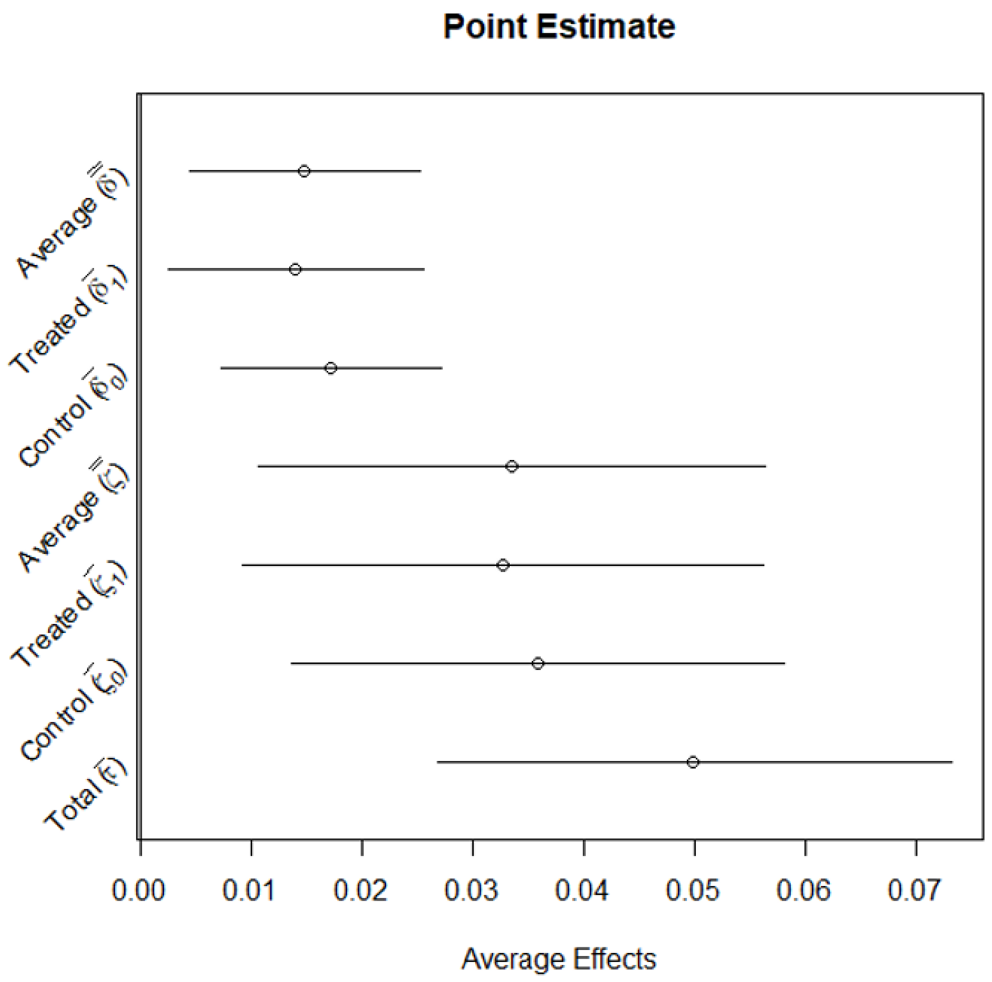

Figure S1: Graphical summary of the results from the 'multimed' function under the homogeneous interaction assumption.

For peer review only - http://bmjopen.bmj.com/site/about/guidelines.xhtml

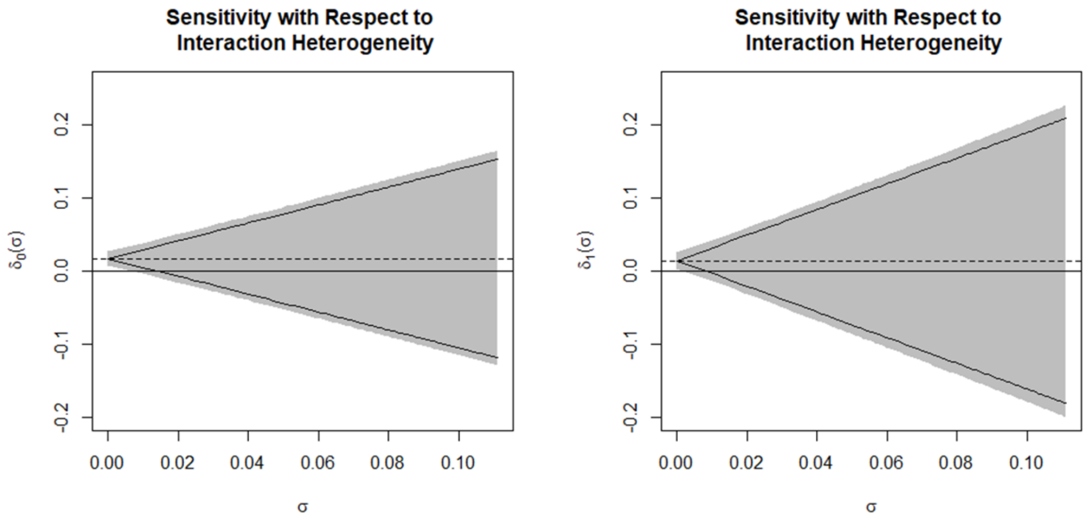

Figure S2: Graphical summary of sensitivity analysis using the 'multimed' function. Results as a function of σ and R^2. For both δ(1) and δ(0), the lower bounds sharply decrease as we increase the value of υ and cross the Zero line at small values of υ [0.011 for .δ(1) and 0.015 for .δ(0)].

For peer review only

STROBE Statement—Checklist of items that should be included in reports of *cross-sectional studies*

	Item No	Recommendation	Page No
**Title and abstract**	1	(*a*) Indicate the study's design with a commonly used term in the title or the abstract	1-2
		(*b*) Provide in the abstract an informative and balanced summary of what was done and what was found	2
**Introduction**			
Background/rationale	2	Explain the scientific background and rationale for the investigation being reported	3-4
Objectives	3	State specific objectives, including any prespecified hypotheses	4
**Methods**			
Study design	4	Present key elements of study design early in the paper	4
Setting	5	Describe the setting, locations, and relevant dates, including periods of recruitment, exposure, follow-up, and data collection	4-5
Participants	6	(*a*) Give the eligibility criteria, and the sources and methods of selection of participants	4-5
Variables	7	Clearly define all outcomes, exposures, predictors, potential confounders, and effect modifiers. Give diagnostic criteria, if applicable	5-7
Data sources/ measurement	8*	For each variable of interest, give sources of data and details of methods of assessment (measurement). Describe comparability of assessment methods if there is more than one group	5-7
Bias	9	Describe any efforts to address potential sources of bias	NA
Study size	10	Explain how the study size was arrived at	4
Quantitative variables	11	Explain how quantitative variables were handled in the analyses. If applicable, describe which groupings were chosen and why	8-9
Statistical methods	12	(*a*) Describe all statistical methods, including those used to control for confounding	8-9
		(*b*) Describe any methods used to examine subgroups and interactions	8-9
		(*c*) Explain how missing data were addressed	NA
		(*d*) If applicable, describe analytical methods taking account of sampling strategy	8-9
		(*e*) Describe any sensitivity analyses	NA
**Results**			
Participants	13*	(a) Report numbers of individuals at each stage of study—eg numbers potentially eligible, examined for eligibility, confirmed eligible, included in the study, completing follow-up, and analysed	10
		(b) Give reasons for non-participation at each stage	NA
		(c) Consider use of a flow diagram	NA
Descriptive data	14*	(a) Give characteristics of study participants (eg demographic, clinical, social) and information on exposures and potential confounders	10
		(b) Indicate number of participants with missing data for each variable of interest	10
Outcome data	15*	Report numbers of outcome events or summary measures	11-12

For peer review only - http://bmjopen.bmj.com/site/about/guidelines.xhtml

Main results	16	(*a*) Give unadjusted estimates and, if applicable, confounder-adjusted estimates and their precision (eg, 95% confidence interval). Make clear which confounders were adjusted for and why they were included	11-12
		(*b*) Report category boundaries when continuous variables were categorized	11-12
		(*c*) If relevant, consider translating estimates of relative risk into absolute risk for a meaningful time period	NA
Other analyses	17	Report other analyses done—eg analyses of subgroups and interactions, and sensitivity analyses	12
**Discussion**			
Key results	18	Summarise key results with reference to study objectives	12-13
Limitations	19	Discuss limitations of the study, taking into account sources of potential bias or imprecision. Discuss both direction and magnitude of any potential bias	14-15
Interpretation	20	Give a cautious overall interpretation of results considering objectives, limitations, multiplicity of analyses, results from similar studies, and other relevant evidence	13-14
Generalisability	21	Discuss the generalisability (external validity) of the study results	13-15
**Other information**			
Funding	22	Give the source of funding and the role of the funders for the present study and, if applicable, for the original study on which the present article is based	16

*Give information separately for exposed and unexposed groups.

Note: An Explanation and Elaboration article discusses each checklist item and gives methodological background and published examples of transparent reporting. The STROBE checklist is best used in conjunction with this article (freely available on the Web sites of PLoS Medicine at http://www.plosmedicine.org/, Annals of Internal Medicine at http://www.annals.org/, and Epidemiology at http://www.epidem.com/). Information on the STROBE Initiative is available at www.strobe-statement.org.

For peer review only

For peer review only - http://bmjopen.bmj.com/site/about/guidelines.xhtml
